# Supplementary material for: Allosteric regulation and crystallographic fragment screening of SARS-CoV-2 NSP15 endoribonuclease
Source: Nucleic Acids Res. 2023 Apr 28;51(10):5255–70. doi: 10.1093/nar/gkad314 (PMC10250223; doi:10.1093/nar/gkad314)
Supplement: gkad314_Supplemental_Files [file gkad314_supplemental_files.zip › Supplementary datasets.zip!/159/Refine_4/01-BUSTER/Cycle-2/LIST.html]

Refining

**BUSTER 2.X**: A suite of programs for macromolecular structure refinement by maximum-likelihood and Bayesian methods  
Copyright © 1992-2020 Global Phasing Ltd.  
All rights reserved.  
Authors: G. Bricogne, E. Blanc, M. Brandl, C. Flensburg, P. Keller, W. Paciorek, P. Roversi, O. Smart, C. Vonrhein, T. Womack  

Please reference use of the software as:

- BUSTER-TNT 2.X, Global Phasing Ltd, Sheraton House, Cambridge CB3 0AX, UK

Version: 2.10.3 for linux64-ifort made on May 20 2020 at 01:14:34  
Licenced to: Diamond Light Source, Scientific Software  

```
---


### Parsing of the BUSTER cardfile

TITLE : autoBUSTER refinement

   Cell information

   Symmetry information

 Using CCP4 X-ray form factors.

   Missing structure chemical composition

 Using ATOM block for chemical composition.


   Shell information

### Parsing of the TNT cardfile


---


### Reading observations file

 Number of reflexions in input:  91725

      Number of accepted reflexions:  85472

      Number of rejected reflexions:      0

   Reflexions in test set:            4245

   Reflexions in working set:        81227


---


### 1. Generating initial model

 2.10 * Shannon sampling rate. NX NY NZ:   360  360  240


  Effective Shannon rates are:    2.43    2.43    2.22


   Allocated BUFFER of size:    119.312 Mb.

   Initial Geometry Information


 No missing atoms declared


   Summary of model volumes

 No missing atoms declared: no prior computed


 Computing bulk solvent model scattering


 NCSMASK PDB radius for solvent mask:     2.15 Angstrom

 running PDBSET (setting cell and spacegroup P1) ... done
 running NCSMASK [1] (creating mask, SMOOTH 0.923692) ... done
 running MAPDUMP (to check if reduction to unit cell is needed) ... done
 running MAPMASK (converting mask to map) ... done

   Generation of babslv by theta-filter

   Electron density in frg mask:  0.420 Å-1

 FOBS  put on rough absolute scale by Wilson plot :
           K        =  0.47072E+02
           B        =     -6.576

 

### Cycle 0001: MaxLik scaling and refinement


   MaxLik Scaling Cycle 0001

   Wilson plot

   Statistics in resolution bins just after scaling
```

|  |  |
| --- | --- |
|  | - Rxpct factors plot- Rxpct factors table- <Log-Lik Gain>- Ln Sigma A- Correlation Coefficients |

```
   <Log-Likelihood> of null-hypothesis

Structure Factor Amplitudes


   Average Figure of Merit

 Switching off refinement of overall temperature factor


 Added B =     0.4429 Å2 to all atoms in fragment


 Added B =     0.4429 Å2 to B_SOLV

```
gelly refinement module
 Authors (2004-2009) O. S. Smart, C. Flensburg, W. Paciorek, C. Vonrhein, 
                     M. Brandl, T. O. Womack and G. Bricogne
         (2010-2011) O. S. Smart, C. Flensburg, W. Paciorek, C. Vonrhein, 
                     T. O. Womack and G. Bricogne
         (2012-2014) O. S. Smart, C. Flensburg, W. Paciorek, C. Vonrhein, 
                     and G. Bricogne
         (2015-2020) G. Bricogne, C. Flensburg, W. Paciorek, C. Vonrhein
 Copyright © 2004-2020 Global Phasing Ltd. 
 All rights reserved.
 gelly module compilation date May 20 2020 01:11:20
 
 gelly uses routines from TNT geometry. References: 
     Tronrud, D. E., Ten Eyck, L. F., Matthews, B. W. (1987)
     "An Efficient General-Purpose Least-Squares Refinement Program 
     for Macromolecular Structures". Acta Crystallogr A, 43:489-501.
     Tronrud, D. E. (1997). "The TNT Refinement Package", 
     in Macromolecular Crystallography, Part B, Eds Charlie Carter 
     and Robert Sweet, Methods in Enzymology, 277:306-319.
 wildcard matching function by Julian Robichaux http://www.nsftools.com

 No -random_seed flag found. Random number stream initialised using:
2147483562
2147483398

 Control flags set to:  -autoncs -hydrogens_input_occ2zero \
                     -hydrogens_output_occ2heavy -screen \
                     100 -screen_sigma \
                     3.0 -keeppdb \
                     1000000 -taomonitor_verbose \
                     2 
 

 ############### Starting setup via TNT routines

 Setting up tnt routines including license check:

                   TNT Stereochemistry Program
 Version: 5.22.3.0
 Licence for Diamond Light Source, Scientific Software is valid
 
 Using TNT geometry routines to load the tnt control file: gelly.tnt
 Using TNT routines to load initial coordinates from file fragment.cor
 Expecting 6118 atoms

 Found     2189 "NOTE BUSTER_RESIDUE_ATOM_DATA" cards
 Have copied information for    6118 atoms from TNT to gelly's store 
 
 The TNT WEIGHT cards used by this run:
 -----WEIGHT       BOND     2.0
 -----WEIGHT       ANGLE    2.0
 -----WEIGHT       IMPROPER 0.0
 -----WEIGHT       TORSION  2.0
 -----WEIGHT       PSEUDO   0.0
 -----WEIGHT       TRIGONAL 2.0
 -----WEIGHT       PLANE    5.0
 -----WEIGHT       CONTACT  5.0
 -----WEIGHT       BCORREL  10.0
 -----WEIGHT       CHIRAL   5.0
 -----WEIGHT       NCS      50.0
 -----WEIGHT       IDEAL    4.0
 -----WEIGHT       RFACTOR  2.21
 
 Dictionary versions picked up from NOTE BUSTER_RESTRAINT_DICT cards as: 
 ------ protgeo_eh99.dat (V1.6.4.17) 20191001  STANDARD AMINO ACID DICTIONARY. BONDS AND ANGLES FROM ENGH AND HUBER EH99. OTHER VALUES BASED ON PREVIOUS TNT OR TAKEN FROM CCP4. INCLUDES HYDROGEN ATOMS.
 ------ exoticaa.dat (V1.3.4.15) 20200327  COLLECTION OF NON-STANDARD AMINO ACIDS, MAINLY EH91 WITHOUT IDEAL DISTANCE INFO
 ------ nuclgeo.dat (V1.13.4.8) 20190909
 ------ bcorrel.dat (V1.15.10.2) 20190616
 ------ contact.dat (V1.15.12.10) 20191121
 ------ idealdist_contact.dat (V1.3.4.12) 20191001  IDEAL-DISTANCE CONTACT TERM DATA AS USED IN PROLSQ. VALUES USED HERE ARE BASED ON DATA HARVESTING OF PDB AS OF 20190109.
 ------ restraints for LIG (Unknown) from cif dictionary Z1437171658.cif
 ------ restraints for CIT (CITRIC ACID) from cif dictionary CIT.cif; generated by GRADE_PDB_LIGAND 1.2.12 (October 4 2016) using MOGUL 1.7.2(RC1), CSD as538be, with quantum mechanics RM1 ((DatabaseID PDB-CIT))
 ------ assume.dat (V1.9.4.2) 20180705
 
 Have picked up spacegroup name for pdb output as 'P 63      '
 Setting up Chain/Residue arrays
 
 Have command line specification -autoncs*
     Have found       1 NCS relations
     Pairwise matching residue number and type between chains: 
                    A        B        S    
          A         .       99%       -   
          B                  .        -   
          S                           .   
       Table key--                                                       
          all   : two chains have 100% match and same number of residues 
         multi  : ditto and they contribute to a multi chain ncs cluster 
          ***%  : two chains have >80% matching residue number/type      
           -    : chains appear to be unrelated                          
      chain A     have identified ncs to     1 other chains. So will weight each autoncs by (       6.666/#)=      6.6660
      chain B     have identified ncs to     1 other chains. So will weight each autoncs by (       6.666/#)=      6.6660
      chain S     have identified ncs to     0 other chains. 
     For .Gelly cards produced see below - after pruning
 
 Setting up BUSTER_SET groups
 Have found    89 BUSTER_SET cards
 Now silently applying automatic/dictionary set definitions. For a verbose listing use '-verbose_set' flag.
 Applying user defined set definitions:
 =====NOTE BUSTER_SET ncsautoXcld = Water --> set 'ncsautoXcld' now has   581 atoms 
 =====NOTE BUSTER_SET ncsautoABset = Chain_A \ ncsautoXcld --> set 'ncsautoABset' now has  2770 atoms 
 Have defined    48 sets: 
      set:                 'All' has   6118 atoms in   1281 residues - automatically defined
      set:               'Empty' has      0 atoms in      0 residues - automatically defined
      set:            'ExoticAA' has      0 atoms in      0 residues - defined by dictionary file
      set:           'ExoticNUC' has      0 atoms in      0 residues - defined by dictionary file
      set:              'NotHET' has   5514 atoms in    698 residues - defined by dictionary file
      set:             'Chain_A' has   2770 atoms in    350 residues - automatically defined
      set:             'Chain_B' has   2767 atoms in    350 residues - automatically defined
      set:             'Chain_S' has    581 atoms in    581 residues - automatically defined
      set:               'Water' has    581 atoms in    581 residues - defined by dictionary file
      set:          'StdProtein' has   5514 atoms in    698 residues - defined by dictionary file
      set:             'Protein' has   5514 atoms in    698 residues - defined by dictionary file
      set:                'Back' has   2794 atoms in    698 residues - defined by dictionary file
      set:                'Side' has   2720 atoms in    650 residues - defined by dictionary file
      set:        'RnaCanonical' has      0 atoms in      0 residues - defined by dictionary file
      set:        'DnaCanonical' has      0 atoms in      0 residues - defined by dictionary file
      set:     'RnaDnaCanonical' has      0 atoms in      0 residues - defined by dictionary file
      set:     'DnaRnaCanonical' has      0 atoms in      0 residues - defined by dictionary file
      set:         'RnaExtended' has      0 atoms in      0 residues - defined by dictionary file
      set:         'DnaExtended' has      0 atoms in      0 residues - defined by dictionary file
      set:      'RnaDnaExtended' has      0 atoms in      0 residues - defined by dictionary file
      set:      'DnaRnaExtended' has      0 atoms in      0 residues - defined by dictionary file
      set:                 'Dna' has      0 atoms in      0 residues - defined by dictionary file
      set:                 'Rna' has      0 atoms in      0 residues - defined by dictionary file
      set:              'RnaDna' has      0 atoms in      0 residues - defined by dictionary file
      set:              'DnaRna' has      0 atoms in      0 residues - defined by dictionary file
      set:             'Nucleic' has      0 atoms in      0 residues - defined by dictionary file
      set:             'Polymer' has   5514 atoms in    698 residues - defined by dictionary file
      set: 'BufferWeichenberger' has     13 atoms in      1 residues - defined by dictionary file
      set:    'BufferAdditional' has      0 atoms in      0 residues - defined by dictionary file
      set:              'Buffer' has     13 atoms in      1 residues - defined by dictionary file
      set:         'MetalAlkali' has      0 atoms in      0 residues - defined by dictionary file
      set:  'MetalAlkalineEarth' has      0 atoms in      0 residues - defined by dictionary file
      set:     'MetalTransition' has      0 atoms in      0 residues - defined by dictionary file
      set: 'MetalPostTransition' has      0 atoms in      0 residues - defined by dictionary file
      set:    'MetalLanthanides' has      0 atoms in      0 residues - defined by dictionary file
      set:               'Metal' has      0 atoms in      0 residues - defined by dictionary file
      set:         'SugarAlphaD' has      0 atoms in      0 residues - defined by dictionary file
      set:         'SugarAlphaL' has      0 atoms in      0 residues - defined by dictionary file
      set:               'Sugar' has      0 atoms in      0 residues - defined by dictionary file
      set:               'Other' has     23 atoms in      2 residues - defined by dictionary file
      set:            'Hydrogen' has      0 atoms in      0 residues - defined by dictionary file
      set:         'nonHydrogen' has   6118 atoms in   1281 residues - defined by dictionary file
      set:             'OccZero' has      0 atoms in      0 residues - defined by dictionary file
      set:          'OccNonZero' has   6118 atoms in   1281 residues - defined by dictionary file
      set:             'OccFull' has   6076 atoms in   1278 residues - defined by dictionary file
      set:          'OccNonFull' has     42 atoms in      3 residues - defined by dictionary file
      set:         'ncsautoXcld' has    581 atoms in    581 residues - user specified
      set:        'ncsautoABset' has   2770 atoms in    350 residues - user specified
 No command line arguments '-occ_allhet' or '-occ_restyp' so will not setup automated occcupancy refinement
 
 Interpreting CONSTANT and FREE cards: 
 =====CONSTANT     OCC
 Have found and interpreted      1 CONSTANT cards in the TNT input. 
 Before any COMBINE - Number of atoms with fixed XYZ =     0
 Before any COMBINE - Number of atoms with fixed B   =     0
 Before any COMBINE - Number of atoms with fixed OCC =  6118
 Before any COMBINE - Number of atoms with fixed BIJ =   581
                            cf total number of atoms =  6118
 
 There are no COMBINE cards to be found in the TNT card input 
 
 There are no NOTE BUSTER_COMBINE_RES cards to be found in the TNT card input 

 ############### Working out full connectivity list


 ############### Working out equivalent atoms


 Residue with most atoms given below:
        A|58:N(TRP)         
        A|58:CA(TRP)        
        A|58:CB(TRP)        
        A|58:CG(TRP)        
        A|58:CD1(TRP)       
        A|58:NE1(TRP)       
        A|58:CE2(TRP)       
        A|58:CD2(TRP)       
        A|58:CE3(TRP)       
        A|58:CZ3(TRP)       
        A|58:CH2(TRP)       
        A|58:CZ2(TRP)       
        A|58:C(TRP)         
        A|58:O(TRP)         


 Have no '-equiv_check_plus' argument - so will take atom type into
 account when checking for equivalence of otherwise identical atoms.
 

 ############### Looking at dihedrals


 ############### Looking at chirals

 NOTE BUSTER_CHIRAL_ANGLECUT card found - read value=    -31.00 degs from it
 NOTE BUSTER_CHIRAL_SIGMA card found - read value=      1.70 degs from it
 RANGE allowed for B variables in refinement:   from      3.00000 to    300.00000

 ############### Preparing for PDB output


 ############### Processing extra restraints explicitly defined

 No 'NOTE BUSTER_TARGET' cards or -target?? args found (used to read coordinates files for soft ncs to external)
 No 'NOTE BUSTER_OCCSUM'     cards found - so not using sum of occupancy restraints
 No 'NOTE BUSTER_DISTANCE'   cards found - so not setting up any additional atom-atom distance restraints
 No 'NOTE BUSTER_UTILANGLE'  cards found - so not setting up any additional restraints on any angle
 No 'NOTE BUSTER_UTILTOR'    cards found - so not setting up any additional sinusoidal restraints on torsions
 
 --- Have some similarity definitions 
     Similarity restraints on RMSD use superposition routines from: 
     Coutsias, E.A., Seok, C., Dill, K.A.(2004)
     "Using quaternions to calculate RMSD", J. Comput. Chem., 25:1849-1857 

 Have found    1 'NOTE BUSTER_SIM_DEFINE' cards 
     Maximum number of chains involved in any SIM group (excluding the template)=     1


 ########## Interpreting card: NOTE BUSTER_SIM_DEFINE ncsautoAB ncsautoABset B
     Number of atoms in template set=  2770
     Number of related chains (other than template) in SIM set=    1
     List of chains to be restrained (excluding template)=   B   
     Pairwise RMSD, XYZ in Angstroms, for SIM group: ncsautoAB
                 A         B   
     A         -----     ----- 
     B         0.670     ----- 
     Overall RMSD,XYZ for the SIM group is     0.670 Angstroms
     Pairwise rms deviation of [individual B's minus their set/chain average] in Angs**2
                 A         B         Bavg
     A         -----     -----     68.61
     B         19.07     -----     58.13
     Overall RMSD,B   for the SIM group is    19.069 Angs**2
     The number of common atoms for the above fits:
                 A         B   
     A         -----     ----- 
     B          2757     ----- 
     (compared to the template set that has    2770 atoms in it)
     List of atoms that are absent in one or more chains for this group: 
                                    A    B   
                    C1      401     +    -
                    O1      401     +    -
                    O2      401     +    -
                    C2      401     +    -
                    C3      401     +    -
                    O7      401     +    -
                    C4      401     +    -
                    C5      401     +    -
                    O3      401     +    -
                    O4      401     +    -
                    C6      401     +    -
                    O5      401     +    -
                    O6      401     +    -
     (key  +  atom present for this chain 
           -  atom absent  for this chain)
 transforms for each of the superpositions orthogonal matrix r11 r12 r13 r21 r22 r23 r31 r32 r33 tx ty tz (PDBSET syntax): 
TRANS_AB -0.878149 -0.478207 -0.013118 -0.478257  0.878220  0.000763  0.011156  0.006944 -0.999914    33.828    32.907    15.258
 reverse transforms 
TRANS_BA -0.878149 -0.478257  0.011156 -0.478207  0.878220  0.006944 -0.013118  0.000763 -0.999914    45.273   -12.829    15.676
 
 Setting up LSSR interatomic distance restraints
 Interpreting card: NOTE BUSTER_SIM_RESTRAIN_LSSR ncsautoAB 6.666
        setting up interatomic distance pair restraints with:
        Dmin=         0.00
        Dmax=         5.50
        sigma=        0.20
        Vmax=         3.00
        weight=       6.67
        number of distance pairs defined for this set is      35461
 Have not supplied either '-sim_swap_equiv' or '-sim_swap_equiv_plus' argument 
   so will not try swaping equivalent atoms to improve NCS and/or target agreement.
 
 NCS by LSSR outlier analysis
 -autoncs option automatic NCS prune routine -------- 
 Residues pruned out by gradient condition:
          A|125     B|125     B|212     B|253
  
 Residues pruned out by function/max condition:
          A|149     A|150     A|173     A|187     A|188     A|215     A|220 
          A|259     A|264     A|345    A|ENDA     B|149     B|150     B|173 
          B|187     B|188     B|215     B|220     B|259     B|264     B|345 
         B|ENDA 
  
 Total number of residues for which LSSR restraints have been pruned out is       26 out of      698 participating
 Number of LSSR restraints pruned out is      1947 this is    5.5% of the total           35461
  
 -autoncs .Gelly cards suitable for adaptation for manual definition/pruning
           (note auto pruning is done in a slightly different way so exact results may differ slightly)
 -autoncs option .Gelly cards also written to file  auto_gelly_cards.txt
NOTE BUSTER_SET ncsautoXcld = Water    # card added - produced by -autoncs
NOTE BUSTER_SET ncsautoXcld = ncsautoXcld + { A|125 A|149 A|150 A|173 A|187 }    # card added - produced by -autoncs
NOTE BUSTER_SET ncsautoXcld = ncsautoXcld + { A|188 A|215 A|220 A|259 A|264 }    # card added - produced by -autoncs
NOTE BUSTER_SET ncsautoXcld = ncsautoXcld + { A|345 A|ENDA }    # card added - produced by -autoncs
NOTE BUSTER_SET ncsautoABset = Chain_A \ ncsautoXcld    # card added - produced by -autoncs
NOTE BUSTER_SIM_DEFINE ncsautoAB ncsautoABset B    # card added - produced by -autoncs
NOTE BUSTER_SIM_RESTRAIN_LSSR ncsautoAB 6.666    # card added - produced by -autoncs

 LSSR function contribution before pruning    126005.5
                             after pruning    108299.1
 Graph of which residues pruned from LSSR: ncs_pruned.mtv
 Graph of LSSR function outliers ncs_function_outliers.mtv
 Graph of LSSR gradient outliers ncs_gradient_outliers.mtv
 LSSR - analysis of distance difference distribution for NCS
         DeltaD less than 0.5 Angs:  proportion of population= 96.66% average DeltaD of subset     0.099 Angs
 Histogram of LSSR deltaD distribution ncs_histogram.mtv
 
 Interpreting card: NOTE BUSTER_TLS_SET tls1 { A|* }
 Interpreting card: NOTE BUSTER_TLS_SET tls2 { B|* }
 Interpreting card: NOTE BUSTER_TLS_SET tls1 { A|* }
 Interpreting card: NOTE BUSTER_TLS_T   tls1     -1.39020E-01 -5.95499E-02  2.01158E-01 -1.38376E-02  3.83768E-02  4.73733E-03
 Interpreting card: NOTE BUSTER_TLS_L   tls1      6.24652E-01  3.28075E-01  9.50159E-02  2.42930E-01 -1.55227E-01  5.25158E-02
 Interpreting card: NOTE BUSTER_TLS_S   tls1      1.76373E-01 -1.14237E-01  1.11924E-01 -5.94811E-02 -8.26876E-02 -2.72786E-02 -4.25898E-02  4.14621E-03
 Interpreting card: NOTE BUSTER_TLS_O   tls1     -2.33295E+01 -8.34950E+01  1.14606E+01
 Interpreting card: NOTE BUSTER_TLS_SET tls2 { B|* }
 Interpreting card: NOTE BUSTER_TLS_T   tls2     -1.42976E-01 -9.83390E-02  2.69975E-01  9.37908E-03  3.33279E-02  5.70942E-03
 Interpreting card: NOTE BUSTER_TLS_L   tls2      5.22272E-01  2.80406E-01  2.72933E-01  2.71705E-01 -2.54092E-01 -2.50673E-01
 Interpreting card: NOTE BUSTER_TLS_S   tls2      1.43174E-03  8.29114E-02 -4.40524E-02  5.30728E-02  1.26218E-01 -2.00028E-02 -3.40003E-02  3.11097E-02
 Interpreting card: NOTE BUSTER_TLS_O   tls2      2.19831E+01 -7.17259E+01  3.45078E+00
 Interpreting card: NOTE BUSTER_TLS_FIX tls1 ALL
 Interpreting card: NOTE BUSTER_TLS_FIX tls2 ALL


 TLS information. Total number of groups:     2


 Group   Natoms    FixRB   FixTLS   Group Tag    Group Spec
     1     2770      Y        Y     tls1    { A|* }
     2     2767      Y        Y     tls2    { B|* }
 
NOTE BUSTER_TLS_SET tls1 { A|* }
NOTE BUSTER_TLS_T   tls1     -1.39020E-01 -5.95499E-02  2.01158E-01 -1.38376E-02  3.83768E-02  4.73733E-03
NOTE BUSTER_TLS_L   tls1      6.24652E-01  3.28075E-01  9.50159E-02  2.42930E-01 -1.55227E-01  5.25158E-02
NOTE BUSTER_TLS_S   tls1      1.76373E-01 -1.14237E-01  1.11924E-01 -5.94811E-02 -8.26876E-02 -2.72786E-02 -4.25898E-02  4.14621E-03
NOTE BUSTER_TLS_O   tls1     -2.33295E+01 -8.34950E+01  1.14606E+01
NOTE BUSTER_TLS_FIX tls1 ALL
 
NOTE BUSTER_TLS_SET tls2 { B|* }
NOTE BUSTER_TLS_T   tls2     -1.42976E-01 -9.83390E-02  2.69975E-01  9.37908E-03  3.33279E-02  5.70942E-03
NOTE BUSTER_TLS_L   tls2      5.22272E-01  2.80406E-01  2.72933E-01  2.71705E-01 -2.54092E-01 -2.50673E-01
NOTE BUSTER_TLS_S   tls2      1.43174E-03  8.29114E-02 -4.40524E-02  5.30728E-02  1.26218E-01 -2.00028E-02 -3.40003E-02  3.11097E-02
NOTE BUSTER_TLS_O   tls2      2.19831E+01 -7.17259E+01  3.45078E+00
NOTE BUSTER_TLS_FIX tls2 ALL


 TLS: mmCIF group specification begin


#
loop_
_pdbx_refine_tls_group.id
_pdbx_refine_tls_group.refine_tls_id
_pdbx_refine_tls_group.beg_PDB_ins_code
_pdbx_refine_tls_group.beg_auth_asym_id
_pdbx_refine_tls_group.beg_auth_seq_id
_pdbx_refine_tls_group.end_PDB_ins_code
_pdbx_refine_tls_group.end_auth_asym_id
_pdbx_refine_tls_group.end_auth_seq_id
_pdbx_refine_tls_group.selection
_pdbx_refine_tls_group.selection_details
   1   1 . A   -1 . A  346 . '{ A|* }'
   2   1 . A  401 . A  401 . '{ A|* }'
   3   2 . B   -1 . B  346 . '{ B|* }'
   4   2 . B  401 . B  401 . '{ B|* }'
#


 TLS: mmCIF group specification end


 Interpreting card: NOTE BUSTER_ANISO_SET Empty
 Interpreting card: NOTE BUSTER_ANISO_WEIGHT SCA  16.0
 Interpreting card: NOTE BUSTER_ANISO_WEIGHT SPH   0.6    0.6
 Interpreting card: NOTE BUSTER_ANISO_WEIGHT SIM   7.8    4.8    2.6    0
 Interpreting card: NOTE BUSTER_ANISO_WEIGHT RIG  21.5   21.5    3.7    0


 After TLS and ANISO: Number of atoms with fixed/combined xyz =     0
 After TLS and ANISO: Number of atoms with fixed/combined B   =     0
 After TLS and ANISO: Number of atoms with fixed/combined OCC =  6118
 After TLS and ANISO: Number of atoms with fixed/combined BIJ =  6118
 After TLS and ANISO: Number of atoms in a TLS group          =  5537
                                     cf total number of atoms =  6118
 

 ############### Setup state vector

 No free_vect_ulim_xyz argument found: using default of  100.000
 No free_vect_llim_xyz argument found: using default of -100.000
 Number of parameters added for TLS description                 =       0
 Number of parameters to describe molecular conformation        =   24472

 ############### Setup free vector

 Number of free parameters to be adjusted in refinement   =   24472

 ############### Setting up own geometry


 No command line argument '-tnt_fn' so will use gelly to do geometry function calculations

 ============= Call TNT functions


 ============= Get TNT symmetry information

 
      Getting symmetry operators from TNT. 
          gelly will classify symmetry using pdb-like convention: 
               SYMOP   SYMMETRY
              NNNMMM   OPERATOR
                1555   X,Y,Z
                2555   -Y,X-Y,Z
                3555   -X+Y,-X,Z
                4555   -X,-Y,1/2+Z
                5555   Y,-X+Y,1/2+Z
                6555   X-Y,X,1/2+Z
          where NNN -> operator number and MMM -> translation vector
 

 ============= Own Gelly geometry/restraints setup


 ----------- Identify bond-types


    Setting up GEOMBONDREC_TYPE_HASH* to be able to map a given bond type (e.g.
    "single") to an internal integer (GBOND_TYPE=1) etc.

    Using default  GEOMBONDREC_TYPE_HASH_STR='SING* 1 DOUB* 2 TRIP* 3 AROM* 4 DELO* 4 METAL* 5':
    to override default use a NOTE BUSTER_GEOMBONDREC_TYPE_HASH_STR gelly card.

 ----------- Classify torsion into peptide omega and other


 ----------- Setup of non-bonded terms

 Ideal-distance contact term setup:
    Restraint function constants (normally these values are from NOTE BUSTER_IDEAL_CONTACT_* cards)
    Sigma for general contacts      0.200 Angs
    Sigma for 1-4 contacts          0.100 Angs (contacts across a torsion)
    Sigma for 1-5 contacts          0.150 Angs
    Sigma for 1-N contacts          0.200 Angs
    Sigma for HBOND pairs           0.100 Angs (contacts between HB_types A and D or B's)
    Contact distance increment for each atom in HBOND          -0.150 Angs 
    Contact distance increment for H-bond acceptor              0.100 Angs 
    Contact distance increment for 1-4 with an O atom          -0.050 Angs 
    Contact distance increment for 1-4 with an N atom          -0.180 Angs 
    Contact distance increment for 1-4 with an C atom          -0.370 Angs 
    Contact distance increment for 1-4 with an S atom          -0.150 Angs 
    Contact distance increment for 1-4 with an P atom          -0.150 Angs 
    Contact distance increment for 1-4 with any other atom     -0.150 Angs 
    All atoms matched for CCP4 atom type.
    Number of CCP4 atom types used is         24 (H-bound and not H-bound counted separately)

 Setting up short/ideal-distance contacts
   Using gelly special position handling. (For TNT instead rerun with argument '-special_tnt')

   No command line argument '-special_all' specified. We will auto-generate
   EXCLUDE cards only for atom-atom contacts across symmetry (i.e. on special
   position) when this involves a contact within a residue to itself.

   Have set up non-bonded exclusions - 1-3 contacts
   Atom A|27:CA         makes the most 1-3 contacts:  6

   Have set up non-bonded exclusions - 1-4 contacts
   Atom A|27:N          makes the most 1-4 contacts:  7

   Have set up non-bonded exclusions - 1-5 contacts
   Atom A|28:C.A        makes the most 1-5 contacts: 10

 ############################################################################################################
 ###################################################### setting up contact terms
 ############################################################################################################


 ============= Call Gelly versions of TNT functions

 WARNING: 1-4 contact without 1-(2,3)-4 data for A|401:O2(CIT)        and A|401:C3(CIT)        X-X
 WARNING: 1-4 contact without 1-(2,3)-4 data for A|401:C2(CIT)        and A|401:O6(CIT)        X-X
 WARNING: 1-4 contact without 1-(2,3)-4 data for B|401:C01(LIG)       and B|401:C04(LIG)       X-X
 WARNING: 1-4 contact without 1-(2,3)-4 data for B|401:N02(LIG)       and B|401:C06(LIG)       X-X

       TERM      weight      number      TNT_fn    Gelly_fn        diff
       BOND       2.000        5652    5571.950    5571.950       0.000
       ANGL       2.000        7670   12501.782   12501.782       0.000
       IMPR       0.000           0       0.000       0.000       0.000
       TORS       2.000        1963    3085.147    3085.147      -0.000
       TRIG       2.000           0       0.000       0.000       0.000
       PLAN       5.000         963   16259.732   16259.732      -0.000
       CONT       5.000           0     149.552       0.000     149.552
       BCOR      10.000        5630  122854.508  125200.291   -2345.783


 ============= Call Gelly geometry functions

   Switching torsion functional form to sinusoidal rather than split harmonic functional.
      if you want this not to happen then rerun with '-torsharm'
      Weight for sinusoidal torsion=     2.000
   BCOR function/gradient will be split between H and non-H atoms (as for any atom pair). Use '-bcorrel_all_in_hydrogens' to change this.

 ----------- Fix B-correlation restraints

 
 Checking for bonds that do not have a bcorrel restraint defined
 We will wipe all existing BCORrels if OCC=0.0 atom(s) is/are involved. This can be' switched off via the '-bcorrel_nowipe_zeroocc' flag.
 We will wipe all existing BCORrels if only non-hydrogen atom(s) with fixed B(iso) are involved. This can be switched off via the '-bcorrel_nowipe_fixb_nonhydrogens' flag.
 We will keep all existing BCORrels if hydrogen atom(s) with fixed B(iso) is/are involved. This can be switched off via the '-bcorrel_wipe_fixb_hydrogens' flag.
 No -bcorrel_missing_sigma found. So will set sigma to     2.5 Ang**2 for any bond with no existing BCORrel
 No -bcorrel_missing_sigma_hydrogens found. So will set sigma to     1.0 Ang**2 for any bond with no existing BCORrel involving hydrogens

 No active BCORels found involving OCC=0.0 atom(s)


 No active BCORels found involving fixed-B (only) non-hydrogen atom(s)

    add a default BCOR for bond A|401:C1(CIT) A|401:C2(CIT)
    add a default BCOR for bond A|401:C1(CIT) A|401:O1(CIT)
    add a default BCOR for bond A|401:C1(CIT) A|401:O2(CIT)
    add a default BCOR for bond A|401:C2(CIT) A|401:C3(CIT)
    add a default BCOR for bond A|401:C3(CIT) A|401:C4(CIT)
    add a default BCOR for bond A|401:C3(CIT) A|401:C6(CIT)
    add a default BCOR for bond A|401:C3(CIT) A|401:O7(CIT)
    add a default BCOR for bond A|401:C4(CIT) A|401:C5(CIT)
    add a default BCOR for bond A|401:C5(CIT) A|401:O3(CIT)
    add a default BCOR for bond A|401:C5(CIT) A|401:O4(CIT)
    add a default BCOR for bond A|401:C6(CIT) A|401:O5(CIT)
    add a default BCOR for bond A|401:C6(CIT) A|401:O6(CIT)
    add a default BCOR for bond B|401:C01(LIG) B|401:N02(LIG)
    add a default BCOR for bond B|401:N02(LIG) B|401:C03(LIG)
    add a default BCOR for bond B|401:C03(LIG) B|401:C04(LIG)
    add a default BCOR for bond B|401:C03(LIG) B|401:C05(LIG)
    add a default BCOR for bond B|401:C05(LIG) B|401:C06(LIG)
    add a default BCOR for bond B|401:C05(LIG) B|401:C10(LIG)
    add a default BCOR for bond B|401:C06(LIG) B|401:N07(LIG)
    add a default BCOR for bond B|401:N07(LIG) B|401:N08(LIG)
    add a default BCOR for bond B|401:N08(LIG) B|401:C09(LIG)
    add a default BCOR for bond B|401:N08(LIG) B|401:C10(LIG)

 Have added a total of        22 BCORrels for bonds which do not already have a BCORrel


 No bonds (for potential BCORrel addition) were skipped due to both atoms having constant/fixed B(iso)


 ----------- Deactivate hydrogen-free planes if hydrogen atoms present

 A total of       0 planes have been deactivated because they are duplicated hydrogen-free and with-hydrogen forms.

 ----------- Handle peptide omega angle(s)

      Torsion angles split into peptide omega and other
         Number of peptide omega angles is       698
         Number of other torsion is             1265
      Peptide omega angles are monitored rather than directly restrained (PLANE used instead).

 ----------- Fix cis-PRO

 Now apply the fix to get correct restraints for cis PRO residues
   Have got      11 bonds records and      26 angles
   from GEOMETRY cards for the special PROC residue
   Weight for ideal-distance contact term=     4.000

 ----------- Prepare SSBOND/LINK for output PDB file


 ----------- Prepare for QM


 ----------- Prepare symmetry in utility distance restraints


 ############### Setting up Rhofit contacts (if needed)

 No ligand-(fixed protein) contact term will be used

 ############### Working out initial function and gradient


   MaxLik Scaling Cycle 0002
 
 Initial functional value = 5.117779E+05 rms gradient=      720.836
 X-ray contribution to function value = 2.136868E+05
 Geometry contribution (inc ncs)      = 2.980911E+05
 Geometry function summary
     term                           number     weight       rms        GooF      function     fn/numb
     ================================================================================================
     BOND bond lengths (angs)       5652       2.000      0.0141       0.702      5571.95       0.986
     ANGL bond angles (degs)        7670       2.000        1.51       0.903     12501.78       1.630
     TORS split-harmonic tors       1265       0.000       17.53       1.104         0.00       0.000
     SINTOR sinusoidal tors         1265       2.000       17.53                  2356.12       1.863
     PLAN general plane (angs)       963       5.000      0.0368       1.838     16259.73      16.884
     BCORrelations (angs^2)         5652      10.000       4.207       1.501    127388.93      22.539
     CONT (bad contacts)               0       5.000                                 0.00
     IDEAL(ideal-dist contact)      5393       4.000                             25706.59
     CHIRAL (gelly semiharmon)       740       5.000                                 6.86
     SIM similarity restraint for NCS and/or TARGET                             108299.11
     OCCSUM restraint on sum of occupanies                                           0.00       as none defined
     DISTAN utility restraints interatomic distance                                  0.00       as none defined
     UTILANGLE utility angle restraints                                              0.00       as none defined
     UTILTOR utility restraints on torsion angles                                    0.00       as none defined
     Peptide omega torsion angles                           7.91 degs - monitor only
 
 WARNING: Have |delta/sigma| deviations > 5.0 sigma. Number of outliers for each term: 
 WARNING:         3 bond angles.  Worst is     8.0 sigs   124.28 degs B|90:NE=CZ=NH1 (ARG)
 WARNING:        12 planes.       Worst is     7.1 sigs     0.14 Angs B|190:C=B|190:CA=B|190:O=B|191:N=B|191:CA
 WARNING:         2 idealD contacts. Worst     5.9 sigs     2.77 Angs B|297:C(LEU)=B|298:C(LEU)
 WARNING:        45 Bcorrel.      Worst is    11.5 sigs    28.86 Angs^2 B|344:C(LYS)=B|345:N(LEU)

 Weighted rms      0.0141         1.515  (temporary rms bond and angle for BUSTER to pick up!)
 Weighted intra-residual rms(bond) for StdProtein (excluding H)      0.0145
 Weighted intra-residual rms(bond) for Buffer     (excluding H)      0.0274

 ############### Running initial check


 Initial Geometry Sanity Check

 Check for duplicate bonds: no duplicates found
 
 Check for duplicate or missing bond angles: all are OK
 Check for bond between an atom and itself not involving symmetry:  all are OK
   There are no bad contacts where Delta/sigma is greater than     6.5
   Worst contact dist=   2.768 std=   3.029 sigma=   0.044 abs(Delta/sigma)=    5.932 ats B|297:C (LEU) B|298:C (LEU)
   It is likely that the geometry libraries, sequence info and links match the input molecule
 GEOMETRY CHECK OVERALL:  PASS


 ############### Creating geometry/screen report


 ############### Option "tao" - run Tao optimizer

 TAO: Toolkit for Advanced Optimization. Reference: 
     Munson, T., Sarich, J., Wild, S., Benson, S., McInnes, L.C. (2018)
     "TAO User Manual (Revision 3.9)", Mathematics and Computer Science
     Division, Argonne National Laboratory
     ANL/MCS-TM-322, http://www.mcs.anl.gov/petsc/
 
 Have found -keeppdb flag so will write out a .pdb file every 1000000 its during opt
 Will apply TAO optimization 
 Maximum number of iterations      100
 Convergence test on Grms       4.0000 (-glim)
 Limit on rms displacement to original position (rmsD2init): NONE test inactive (-dlim)

    analysing argument no 0 : -autoncs with j=0
    analysing argument no 1 : -hydrogens_input_occ2zero with j=0
    analysing argument no 2 : -hydrogens_output_occ2heavy with j=0
    analysing argument no 3 : -screen with j=0
    analysing argument no 4 : 100 with j=0
    analysing argument no 5 : -screen_sigma with j=0
    analysing argument no 6 : 3.0 with j=0
    analysing argument no 7 : -keeppdb with j=0
    analysing argument no 8 : 1000000 with j=0
    analysing argument no 9 : -taomonitor_verbose with j=0
    found argument starting with "-tao" : -taomonitor_verbose
    analysing argument no 10 : 2 with j=1
    found value after -tao/-options_/-viewer_ argument : "2"
 PETSC/TAO: setting option "-taomonitor_verbose" = "2"


   MaxLik Scaling Cycle 0003
   Iters     Tot_Funct    Xray_Funct    Geom_Funct          Grms     rmsD2init     maxD2init             R         Rfree           LLG       LLGfree     rms(bond)    rms(angle)       Opttag_H
   Iters             K   B_IMPF_FRAG        K_MISS   B_TEMP_MISS   K_IMPF_MISS   B_IMPF_MISS        K_SOLV   B_TEMP_SOLV   K_IMPF_SOLV   B_IMPF_SOLV        B_TEMP     B_TEMP_11     B_TEMP_12     B_TEMP_22     B_TEMP_13     B_TEMP_23     B_TEMP_33      K_WILSON      B_WILSON      FGCYCLES       Scatag_H
   Iters          BOND         ANGLE      IMPROPER       TORSION    SINTORSION      TRIGONAL        PLANAR  BCORRELATION       CONTACT     IDEALDIST        CHIRAL    SIMILARITY       OCCSSUM  UTILDISTANCE     UTILANGLE   UTILTORSION        RHOFIT        ADP-01        ADP-02        ADP-03        ADP-04        ADP-05        ADP-06        ADP-07        ADP-08        ADP-09        ADP-10      RHOFITPC            QM       Funtag_H
       0  5.117779E+05  2.136868E+05  2.980911E+05       720.836  0.000000E+00  0.000000E+00      0.210443      0.221597  0.000000E+00  0.000000E+00     0.0140881       1.51463       Opttag_D
       0       51.4819       2.17453       1.00000  0.000000E+00       1.00000  0.000000E+00      0.820163       59.1348       1.00000       24.4672  0.000000E+00       1.05032  0.000000E+00       1.05032  0.000000E+00  0.000000E+00      -2.10064       52.1585       1.51622        2       Scatag_D
       0       5571.95       12501.8  0.000000E+00  0.000000E+00       2356.12  0.000000E+00       16259.7  1.273889E+05  0.000000E+00       25706.6       6.85875  1.082991E+05 -1.000000E+10 -1.000000E+10 -1.000000E+10 -1.000000E+10 -1.000000E+10  0.000000E+00  0.000000E+00  0.000000E+00  0.000000E+00  0.000000E+00  0.000000E+00  0.000000E+00  0.000000E+00  0.000000E+00  0.000000E+00  0.000000E+00  0.000000E+00       Funtag_D
   Geometry Restraint Deviations SCREEN Information

 

   MaxLik Scaling Cycle 0004

   MaxLik Scaling Cycle 0005

   Wilson plot

   Statistics in resolution bins after refinement cycle 0001
```


|  |  |
| --- | --- |
|  | - Rxpct factors plot- Rxpct factors table- <Log-Lik Gain>- Ln Sigma A- Correlation Coefficients |


```
   Iters     Tot_Funct    Xray_Funct    Geom_Funct          Grms     rmsD2init     maxD2init             R         Rfree           LLG       LLGfree     rms(bond)    rms(angle)
   Iters             K   B_IMPF_FRAG        K_MISS   B_TEMP_MISS   K_IMPF_MISS   B_IMPF_MISS        K_SOLV   B_TEMP_SOLV   K_IMPF_SOLV   B_IMPF_SOLV        B_TEMP     B_TEMP_11     B_TEMP_12     B_TEMP_22     B_TEMP_13     B_TEMP_23     B_TEMP_33      K_WILSON      B_WILSON      FGCYCLES
   Iters          BOND         ANGLE      IMPROPER       TORSION    SINTORSION      TRIGONAL        PLANAR  BCORRELATION       CONTACT     IDEALDIST        CHIRAL    SIMILARITY       OCCSSUM  UTILDISTANCE     UTILANGLE   UTILTORSION        RHOFIT        ADP-01        ADP-02        ADP-03        ADP-04        ADP-05        ADP-06        ADP-07        ADP-08        ADP-09        ADP-10      RHOFITPC            QM
       1  4.333705E+05  2.145807E+05  2.187898E+05       462.699    0.00669751     0.0884060      0.211632      0.223072   -0.00498054   -0.00615096     0.0111064       1.38708       Opttag_D
       1       51.3787       2.22756       1.00000  0.000000E+00       1.00000  0.000000E+00      0.820957       58.9331       1.00000       24.0519  0.000000E+00       1.29017  0.000000E+00       1.29017  0.000000E+00  0.000000E+00      -2.58034       52.1585       1.51622        2       Scatag_D
       1       3462.94       10484.9  0.000000E+00  0.000000E+00       2343.93  0.000000E+00       14240.8       64340.3  0.000000E+00       23881.1       9.49561  1.000263E+05 -1.000000E+10 -1.000000E+10 -1.000000E+10 -1.000000E+10 -1.000000E+10  0.000000E+00  0.000000E+00  0.000000E+00  0.000000E+00  0.000000E+00  0.000000E+00  0.000000E+00  0.000000E+00  0.000000E+00  0.000000E+00  0.000000E+00  0.000000E+00       Funtag_D
   Geometry Restraint Deviations SCREEN Information

 

   MaxLik Scaling Cycle 0006

   Wilson plot

   Statistics in resolution bins after refinement cycle 0002
```


|  |  |
| --- | --- |
|  | - Rxpct factors plot- Rxpct factors table- <Log-Lik Gain>- Ln Sigma A- Correlation Coefficients |


```
   Iters     Tot_Funct    Xray_Funct    Geom_Funct          Grms     rmsD2init     maxD2init             R         Rfree           LLG       LLGfree     rms(bond)    rms(angle)
   Iters             K   B_IMPF_FRAG        K_MISS   B_TEMP_MISS   K_IMPF_MISS   B_IMPF_MISS        K_SOLV   B_TEMP_SOLV   K_IMPF_SOLV   B_IMPF_SOLV        B_TEMP     B_TEMP_11     B_TEMP_12     B_TEMP_22     B_TEMP_13     B_TEMP_23     B_TEMP_33      K_WILSON      B_WILSON      FGCYCLES
   Iters          BOND         ANGLE      IMPROPER       TORSION    SINTORSION      TRIGONAL        PLANAR  BCORRELATION       CONTACT     IDEALDIST        CHIRAL    SIMILARITY       OCCSSUM  UTILDISTANCE     UTILANGLE   UTILTORSION        RHOFIT        ADP-01        ADP-02        ADP-03        ADP-04        ADP-05        ADP-06        ADP-07        ADP-08        ADP-09        ADP-10      RHOFITPC            QM
       2  4.011090E+05  2.148901E+05  1.862189E+05       330.337     0.0117201     0.0515829      0.212162      0.223869   -0.00670411   -0.00840879    0.00942688       1.28308       Opttag_D
       2       51.3144       2.24102       1.00000  0.000000E+00       1.00000  0.000000E+00      0.821306       58.6256       1.00000       23.9665  0.000000E+00       1.40799  0.000000E+00       1.40799  0.000000E+00  0.000000E+00      -2.81597       52.1585       1.51622        1       Scatag_D
       2       2494.80       8971.54  0.000000E+00  0.000000E+00       2332.75  0.000000E+00       12665.8       44621.7  0.000000E+00       22540.1       21.3090       92570.9 -1.000000E+10 -1.000000E+10 -1.000000E+10 -1.000000E+10 -1.000000E+10  0.000000E+00  0.000000E+00  0.000000E+00  0.000000E+00  0.000000E+00  0.000000E+00  0.000000E+00  0.000000E+00  0.000000E+00  0.000000E+00  0.000000E+00  0.000000E+00       Funtag_D
   Geometry Restraint Deviations SCREEN Information

 
       3  3.722665E+05  2.155140E+05  1.567525E+05       269.254     0.0218078     0.0940905      0.213225      0.225284    -0.0101793    -0.0124628     0.0107636       1.18425       Opttag_D
       3       51.2397       2.27687       1.00000  0.000000E+00       1.00000  0.000000E+00      0.822051       58.1149       1.00000       23.8428  0.000000E+00       1.58875  0.000000E+00       1.58875  0.000000E+00  0.000000E+00      -3.17749       52.1585       1.51622        1       Scatag_D
       3       3252.51       7642.77  0.000000E+00  0.000000E+00       2314.97  0.000000E+00       10163.5       33556.8  0.000000E+00       20501.5       66.2893       79254.1 -1.000000E+10 -1.000000E+10 -1.000000E+10 -1.000000E+10 -1.000000E+10  0.000000E+00  0.000000E+00  0.000000E+00  0.000000E+00  0.000000E+00  0.000000E+00  0.000000E+00  0.000000E+00  0.000000E+00  0.000000E+00  0.000000E+00  0.000000E+00       Funtag_D
       4  3.548694E+05  2.160620E+05  1.388075E+05       306.048     0.0317183      0.131649      0.214209      0.226360    -0.0132321    -0.0157483     0.0126763       1.34046       Opttag_D
       4       51.1796       2.30761       1.00000  0.000000E+00       1.00000  0.000000E+00      0.822617       57.6448       1.00000       23.7983  0.000000E+00       1.71960  0.000000E+00       1.71960  0.000000E+00  0.000000E+00      -3.43920       52.1585       1.51622        1       Scatag_D
       4       4511.13       9791.87  0.000000E+00  0.000000E+00       2301.62  0.000000E+00       8290.68       27680.6  0.000000E+00       19049.8       121.584       67060.1 -1.000000E+10 -1.000000E+10 -1.000000E+10 -1.000000E+10 -1.000000E+10  0.000000E+00  0.000000E+00  0.000000E+00  0.000000E+00  0.000000E+00  0.000000E+00  0.000000E+00  0.000000E+00  0.000000E+00  0.000000E+00  0.000000E+00  0.000000E+00       Funtag_D
       5  3.398748E+05  2.163875E+05  1.234872E+05       225.454     0.0396757      0.157382      0.214871      0.227080    -0.0150457    -0.0177780     0.0124570       1.25686       Opttag_D
       5       51.1273       2.32045       1.00000  0.000000E+00       1.00000  0.000000E+00      0.822871       57.2211       1.00000       23.8655  0.000000E+00       1.79643  0.000000E+00       1.79643  0.000000E+00  0.000000E+00      -3.59285       52.1585       1.51622        1       Scatag_D
       5       4356.39       8608.62  0.000000E+00  0.000000E+00       2293.64  0.000000E+00       7190.67       24132.6  0.000000E+00       18176.6       151.242       58577.5 -1.000000E+10 -1.000000E+10 -1.000000E+10 -1.000000E+10 -1.000000E+10  0.000000E+00  0.000000E+00  0.000000E+00  0.000000E+00  0.000000E+00  0.000000E+00  0.000000E+00  0.000000E+00  0.000000E+00  0.000000E+00  0.000000E+00  0.000000E+00       Funtag_D
       6  3.255719E+05  2.167973E+05  1.087746E+05       213.979     0.0508228      0.186782      0.215742      0.227940    -0.0173287    -0.0203972     0.0114495       1.29068       Opttag_D
       6       51.0570       2.33413       1.00000  0.000000E+00       1.00000  0.000000E+00      0.823073       56.6507       1.00000       24.0168  0.000000E+00       1.88101  0.000000E+00       1.88101  0.000000E+00  0.000000E+00      -3.76201       52.1585       1.51622        1       Scatag_D
       6       3680.19       9078.18  0.000000E+00  0.000000E+00       2286.17  0.000000E+00       6132.30       20529.3  0.000000E+00       17290.9       160.688       49616.8 -1.000000E+10 -1.000000E+10 -1.000000E+10 -1.000000E+10 -1.000000E+10  0.000000E+00  0.000000E+00  0.000000E+00  0.000000E+00  0.000000E+00  0.000000E+00  0.000000E+00  0.000000E+00  0.000000E+00  0.000000E+00  0.000000E+00  0.000000E+00       Funtag_D
       7  3.165740E+05  2.170899E+05       99484.1       201.065     0.0583013      0.201077      0.216354      0.228565    -0.0189586    -0.0223697     0.0105114       1.30500       Opttag_D
       7       51.0075       2.34482       1.00000  0.000000E+00       1.00000  0.000000E+00      0.823153       56.3135       1.00000       24.0997  0.000000E+00       1.93186  0.000000E+00       1.93186  0.000000E+00  0.000000E+00      -3.86373       52.1585       1.51622        1       Scatag_D
       7       3101.86       9280.71  0.000000E+00  0.000000E+00       2283.20  0.000000E+00       5658.51       17334.5  0.000000E+00       16802.1       126.348       44896.9 -1.000000E+10 -1.000000E+10 -1.000000E+10 -1.000000E+10 -1.000000E+10  0.000000E+00  0.000000E+00  0.000000E+00  0.000000E+00  0.000000E+00  0.000000E+00  0.000000E+00  0.000000E+00  0.000000E+00  0.000000E+00  0.000000E+00  0.000000E+00       Funtag_D
       8  3.097759E+05  2.172632E+05       92512.7       161.932     0.0635382      0.214166      0.216745      0.229000    -0.0199236    -0.0235690    0.00977976       1.27511       Opttag_D
       8       50.9689       2.35005       1.00000  0.000000E+00       1.00000  0.000000E+00      0.823174       56.0816       1.00000       24.1413  0.000000E+00       1.96190  0.000000E+00       1.96190  0.000000E+00  0.000000E+00      -3.92381       52.1585       1.51622        1       Scatag_D
       8       2685.08       8860.49  0.000000E+00  0.000000E+00       2279.44  0.000000E+00       5405.34       15074.2  0.000000E+00       16466.2       88.2361       41653.7 -1.000000E+10 -1.000000E+10 -1.000000E+10 -1.000000E+10 -1.000000E+10  0.000000E+00  0.000000E+00  0.000000E+00  0.000000E+00  0.000000E+00  0.000000E+00  0.000000E+00  0.000000E+00  0.000000E+00  0.000000E+00  0.000000E+00  0.000000E+00       Funtag_D
       9  3.044546E+05  2.173634E+05       87091.2       133.174     0.0674651      0.252894      0.217007      0.229331    -0.0204820    -0.0243337    0.00916585       1.22898       Opttag_D
       9       50.9365       2.35182       1.00000  0.000000E+00       1.00000  0.000000E+00      0.823179       55.8973       1.00000       24.1511  0.000000E+00       1.98227  0.000000E+00       1.98227  0.000000E+00  0.000000E+00      -3.96454       52.1585       1.51622        1       Scatag_D
       9       2358.55       8231.01  0.000000E+00  0.000000E+00       2275.36  0.000000E+00       5237.24       13860.8  0.000000E+00       16187.7       66.5278       38874.0 -1.000000E+10 -1.000000E+10 -1.000000E+10 -1.000000E+10 -1.000000E+10  0.000000E+00  0.000000E+00  0.000000E+00  0.000000E+00  0.000000E+00  0.000000E+00  0.000000E+00  0.000000E+00  0.000000E+00  0.000000E+00  0.000000E+00  0.000000E+00       Funtag_D
      10  2.999125E+05  2.174808E+05       82431.7       126.983     0.0713127      0.289669      0.217298      0.229710    -0.0211362    -0.0252164    0.00874852       1.21163       Opttag_D
      10       50.9071       2.35546       1.00000  0.000000E+00       1.00000  0.000000E+00      0.823225       55.6911       1.00000       24.1590  0.000000E+00       2.00315  0.000000E+00       2.00315  0.000000E+00  0.000000E+00      -4.00629       52.1585       1.51622        1       Scatag_D
      10       2148.67       8000.17  0.000000E+00  0.000000E+00       2271.72  0.000000E+00       5032.83       12504.1  0.000000E+00       15896.8       65.9951       36511.3 -1.000000E+10 -1.000000E+10 -1.000000E+10 -1.000000E+10 -1.000000E+10  0.000000E+00  0.000000E+00  0.000000E+00  0.000000E+00  0.000000E+00  0.000000E+00  0.000000E+00  0.000000E+00  0.000000E+00  0.000000E+00  0.000000E+00  0.000000E+00       Funtag_D
      11  2.956257E+05  2.177505E+05       77875.3       169.278     0.0785819      0.347779      0.217910      0.230495    -0.0226381    -0.0269828    0.00865080       1.22380       Opttag_D
      11       50.8619       2.36745       1.00000  0.000000E+00       1.00000  0.000000E+00      0.823356       55.2992       1.00000       24.1903  0.000000E+00       2.04151  0.000000E+00       2.04151  0.000000E+00  0.000000E+00      -4.08301       52.1585       1.51622        1       Scatag_D
      11       2100.94       8161.67  0.000000E+00  0.000000E+00       2265.54  0.000000E+00       4663.46       11609.9  0.000000E+00       15437.1       73.8740       33562.8 -1.000000E+10 -1.000000E+10 -1.000000E+10 -1.000000E+10 -1.000000E+10  0.000000E+00  0.000000E+00  0.000000E+00  0.000000E+00  0.000000E+00  0.000000E+00  0.000000E+00  0.000000E+00  0.000000E+00  0.000000E+00  0.000000E+00  0.000000E+00       Funtag_D
      12  2.918603E+05  2.178716E+05       73988.8       109.901     0.0815893      0.363360      0.218191      0.230799    -0.0233128    -0.0277696    0.00836065       1.17228       Opttag_D
      12       50.8373       2.37144       1.00000  0.000000E+00       1.00000  0.000000E+00      0.823407       55.0865       1.00000       24.2181  0.000000E+00       2.06365  0.000000E+00       2.06365  0.000000E+00  0.000000E+00      -4.12730       52.1585       1.51622        1       Scatag_D
      12       1962.37       7488.96  0.000000E+00  0.000000E+00       2263.66  0.000000E+00       4437.67       10232.9  0.000000E+00       15216.5       78.6895       32308.0 -1.000000E+10 -1.000000E+10 -1.000000E+10 -1.000000E+10 -1.000000E+10  0.000000E+00  0.000000E+00  0.000000E+00  0.000000E+00  0.000000E+00  0.000000E+00  0.000000E+00  0.000000E+00  0.000000E+00  0.000000E+00  0.000000E+00  0.000000E+00       Funtag_D
      13  2.892118E+05  2.179574E+05       71254.4       96.6335     0.0835353      0.366061      0.218393      0.230990    -0.0237910    -0.0283222    0.00821487       1.16231       Opttag_D
      13       50.8164       2.37316       1.00000  0.000000E+00       1.00000  0.000000E+00      0.823424       54.9225       1.00000       24.2471  0.000000E+00       2.08296  0.000000E+00       2.08296  0.000000E+00  0.000000E+00      -4.16593       52.1585       1.51622        1       Scatag_D
      13       1894.53       7362.09  0.000000E+00  0.000000E+00       2262.05  0.000000E+00       4246.87       9081.17  0.000000E+00       15047.2       78.6911       31281.8 -1.000000E+10 -1.000000E+10 -1.000000E+10 -1.000000E+10 -1.000000E+10  0.000000E+00  0.000000E+00  0.000000E+00  0.000000E+00  0.000000E+00  0.000000E+00  0.000000E+00  0.000000E+00  0.000000E+00  0.000000E+00  0.000000E+00  0.000000E+00       Funtag_D
      14  2.863014E+05  2.182292E+05       68072.2       138.709     0.0888477      0.371608      0.218969      0.231547    -0.0253050    -0.0299453    0.00831999       1.17866       Opttag_D
      14       50.7727       2.38424       1.00000  0.000000E+00       1.00000  0.000000E+00      0.823491       54.5549       1.00000       24.3021  0.000000E+00       2.12916  0.000000E+00       2.12916  0.000000E+00  0.000000E+00      -4.25833       52.1585       1.51622        1       Scatag_D
      14       1943.33       7570.76  0.000000E+00  0.000000E+00       2258.28  0.000000E+00       3865.99       8398.84  0.000000E+00       14667.4       76.6066       29290.9 -1.000000E+10 -1.000000E+10 -1.000000E+10 -1.000000E+10 -1.000000E+10  0.000000E+00  0.000000E+00  0.000000E+00  0.000000E+00  0.000000E+00  0.000000E+00  0.000000E+00  0.000000E+00  0.000000E+00  0.000000E+00  0.000000E+00  0.000000E+00       Funtag_D
      15  2.840432E+05  2.184916E+05       65551.6       119.682     0.0934377      0.374758      0.219512      0.232069    -0.0267670    -0.0314949    0.00824342       1.15569       Opttag_D
      15       50.7423       2.39788       1.00000  0.000000E+00       1.00000  0.000000E+00      0.823568       54.2745       1.00000       24.3360  0.000000E+00       2.16698  0.000000E+00       2.16698  0.000000E+00  0.000000E+00      -4.33397       52.1585       1.51622        1       Scatag_D
      15       1907.72       7278.53  0.000000E+00  0.000000E+00       2257.29  0.000000E+00       3625.50       7890.42  0.000000E+00       14375.1       71.7135       28145.3 -1.000000E+10 -1.000000E+10 -1.000000E+10 -1.000000E+10 -1.000000E+10  0.000000E+00  0.000000E+00  0.000000E+00  0.000000E+00  0.000000E+00  0.000000E+00  0.000000E+00  0.000000E+00  0.000000E+00  0.000000E+00  0.000000E+00  0.000000E+00       Funtag_D
      16  2.823015E+05  2.185176E+05       63783.9       82.3355     0.0940258      0.378300      0.219573      0.232133    -0.0269114    -0.0316815    0.00793318       1.13128       Opttag_D
      16       50.7346       2.39821       1.00000  0.000000E+00       1.00000  0.000000E+00      0.823552       54.2213       1.00000       24.3374  0.000000E+00       2.17346  0.000000E+00       2.17346  0.000000E+00  0.000000E+00      -4.34692       52.1585       1.51622        1       Scatag_D
      16       1766.83       6974.27  0.000000E+00  0.000000E+00       2256.14  0.000000E+00       3589.82       7184.59  0.000000E+00       14286.7       66.6061       27659.0 -1.000000E+10 -1.000000E+10 -1.000000E+10 -1.000000E+10 -1.000000E+10  0.000000E+00  0.000000E+00  0.000000E+00  0.000000E+00  0.000000E+00  0.000000E+00  0.000000E+00  0.000000E+00  0.000000E+00  0.000000E+00  0.000000E+00  0.000000E+00       Funtag_D
      17  2.798670E+05  2.186634E+05       61203.6       75.8432     0.0968848      0.394418      0.219892      0.232464    -0.0277239    -0.0325653    0.00768110       1.11731       Opttag_D
      17       50.7142       2.40505       1.00000  0.000000E+00       1.00000  0.000000E+00      0.823558       54.0229       1.00000       24.3433  0.000000E+00       2.19649  0.000000E+00       2.19649  0.000000E+00  0.000000E+00      -4.39297       52.1585       1.51622        1       Scatag_D
      17       1656.33       6803.13  0.000000E+00  0.000000E+00       2253.03  0.000000E+00       3467.30       6506.00  0.000000E+00       13998.5       61.6138       26457.7 -1.000000E+10 -1.000000E+10 -1.000000E+10 -1.000000E+10 -1.000000E+10  0.000000E+00  0.000000E+00  0.000000E+00  0.000000E+00  0.000000E+00  0.000000E+00  0.000000E+00  0.000000E+00  0.000000E+00  0.000000E+00  0.000000E+00  0.000000E+00       Funtag_D
      18  2.786069E+05  2.188598E+05       59747.1       113.334      0.100615      0.413655      0.220313      0.232895    -0.0288180    -0.0336440    0.00774651       1.13182       Opttag_D
      18       50.6974       2.41619       1.00000  0.000000E+00       1.00000  0.000000E+00      0.823610       53.7870       1.00000       24.3621  0.000000E+00       2.22296  0.000000E+00       2.22296  0.000000E+00  0.000000E+00      -4.44591       52.1585       1.51622        1       Scatag_D
      18       1684.66       6980.99  0.000000E+00  0.000000E+00       2251.03  0.000000E+00       3336.52       6025.77  0.000000E+00       13702.9       62.7670       25702.4 -1.000000E+10 -1.000000E+10 -1.000000E+10 -1.000000E+10 -1.000000E+10  0.000000E+00  0.000000E+00  0.000000E+00  0.000000E+00  0.000000E+00  0.000000E+00  0.000000E+00  0.000000E+00  0.000000E+00  0.000000E+00  0.000000E+00  0.000000E+00       Funtag_D
      19  2.771537E+05  2.189487E+05       58204.9       71.4142      0.102416      0.420876      0.220501      0.233082    -0.0293134    -0.0340993    0.00758599       1.10028       Opttag_D
      19       50.6918       2.42141       1.00000  0.000000E+00       1.00000  0.000000E+00      0.823634       53.6645       1.00000       24.3670  0.000000E+00       2.23597  0.000000E+00       2.23597  0.000000E+00  0.000000E+00      -4.47194       52.1585       1.51622        1       Scatag_D
      19       1615.57       6597.32  0.000000E+00  0.000000E+00       2249.02  0.000000E+00       3278.60       5615.67  0.000000E+00       13528.1       64.9740       25255.7 -1.000000E+10 -1.000000E+10 -1.000000E+10 -1.000000E+10 -1.000000E+10  0.000000E+00  0.000000E+00  0.000000E+00  0.000000E+00  0.000000E+00  0.000000E+00  0.000000E+00  0.000000E+00  0.000000E+00  0.000000E+00  0.000000E+00  0.000000E+00       Funtag_D
      20  2.752929E+05  2.190834E+05       56209.5       63.2983      0.105234      0.438939      0.220792      0.233345    -0.0300638    -0.0347128    0.00744500       1.08115       Opttag_D
      20       50.6882       2.43018       1.00000  0.000000E+00       1.00000  0.000000E+00      0.823681       53.4700       1.00000       24.3637  0.000000E+00       2.25852  0.000000E+00       2.25852  0.000000E+00  0.000000E+00      -4.51705       52.1585       1.51622        1       Scatag_D
      20       1556.07       6369.85  0.000000E+00  0.000000E+00       2244.36  0.000000E+00       3188.65       5134.39  0.000000E+00       13210.5       70.0298       24435.6 -1.000000E+10 -1.000000E+10 -1.000000E+10 -1.000000E+10 -1.000000E+10  0.000000E+00  0.000000E+00  0.000000E+00  0.000000E+00  0.000000E+00  0.000000E+00  0.000000E+00  0.000000E+00  0.000000E+00  0.000000E+00  0.000000E+00  0.000000E+00       Funtag_D
      21  2.743503E+05  2.192073E+05       55143.0       102.309      0.107547      0.451121      0.221044      0.233561    -0.0307535    -0.0352855    0.00747142       1.09280       Opttag_D
      21       50.6882       2.43932       1.00000  0.000000E+00       1.00000  0.000000E+00      0.823735       53.3268       1.00000       24.3503  0.000000E+00       2.27872  0.000000E+00       2.27872  0.000000E+00  0.000000E+00      -4.55743       52.1585       1.51622        1       Scatag_D
      21       1567.14       6507.98  0.000000E+00  0.000000E+00       2241.62  0.000000E+00       3122.18       4809.85  0.000000E+00       12958.9       68.5420       23866.8 -1.000000E+10 -1.000000E+10 -1.000000E+10 -1.000000E+10 -1.000000E+10  0.000000E+00  0.000000E+00  0.000000E+00  0.000000E+00  0.000000E+00  0.000000E+00  0.000000E+00  0.000000E+00  0.000000E+00  0.000000E+00  0.000000E+00  0.000000E+00       Funtag_D
      22  2.732695E+05  2.192600E+05       54009.6       67.7950      0.108624      0.476439      0.221152      0.233656    -0.0310472    -0.0355359    0.00735998       1.06565       Opttag_D
      22       50.6896       2.44342       1.00000  0.000000E+00       1.00000  0.000000E+00      0.823759       53.2580       1.00000       24.3388  0.000000E+00       2.28902  0.000000E+00       2.28902  0.000000E+00  0.000000E+00      -4.57803       52.1585       1.51622        1       Scatag_D
      22       1520.74       6188.61  0.000000E+00  0.000000E+00       2239.54  0.000000E+00       3085.65       4603.87  0.000000E+00       12818.8       65.3754       23487.0 -1.000000E+10 -1.000000E+10 -1.000000E+10 -1.000000E+10 -1.000000E+10  0.000000E+00  0.000000E+00  0.000000E+00  0.000000E+00  0.000000E+00  0.000000E+00  0.000000E+00  0.000000E+00  0.000000E+00  0.000000E+00  0.000000E+00  0.000000E+00       Funtag_D
      23  2.717457E+05  2.193378E+05       52408.0       56.7541      0.110463      0.529794      0.221307      0.233846    -0.0314805    -0.0359561    0.00725253       1.04798       Opttag_D
      23       50.6941       2.44999       1.00000  0.000000E+00       1.00000  0.000000E+00      0.823798       53.1417       1.00000       24.3117  0.000000E+00       2.30529  0.000000E+00       2.30529  0.000000E+00  0.000000E+00      -4.61058       52.1585       1.51622        1       Scatag_D
      23       1476.66       5985.02  0.000000E+00  0.000000E+00       2236.31  0.000000E+00       3020.39       4328.70  0.000000E+00       12556.9       56.4437       22747.5 -1.000000E+10 -1.000000E+10 -1.000000E+10 -1.000000E+10 -1.000000E+10  0.000000E+00  0.000000E+00  0.000000E+00  0.000000E+00  0.000000E+00  0.000000E+00  0.000000E+00  0.000000E+00  0.000000E+00  0.000000E+00  0.000000E+00  0.000000E+00       Funtag_D
      24  2.708635E+05  2.194039E+05       51459.6       81.9501      0.112169      0.573895      0.221445      0.234067    -0.0318491    -0.0364337    0.00718669       1.05332       Opttag_D
      24       50.6983       2.45551       1.00000  0.000000E+00       1.00000  0.000000E+00      0.823837       53.0383       1.00000       24.2955  0.000000E+00       2.31551  0.000000E+00       2.31551  0.000000E+00  0.000000E+00      -4.63102       52.1585       1.51622        1       Scatag_D
      24       1449.96       6046.23  0.000000E+00  0.000000E+00       2235.40  0.000000E+00       2972.23       4092.82  0.000000E+00       12352.9       52.3097       22257.8 -1.000000E+10 -1.000000E+10 -1.000000E+10 -1.000000E+10 -1.000000E+10  0.000000E+00  0.000000E+00  0.000000E+00  0.000000E+00  0.000000E+00  0.000000E+00  0.000000E+00  0.000000E+00  0.000000E+00  0.000000E+00  0.000000E+00  0.000000E+00       Funtag_D
      25  2.698938E+05  2.194533E+05       50440.5       56.6925      0.113640      0.609056      0.221543      0.234254    -0.0321241    -0.0368655    0.00704032       1.03468       Opttag_D
      25       50.7027       2.45975       1.00000  0.000000E+00       1.00000  0.000000E+00      0.823871       52.9521       1.00000       24.2857  0.000000E+00       2.32127  0.000000E+00       2.32127  0.000000E+00  0.000000E+00      -4.64254       52.1585       1.51622        1       Scatag_D
      25       1391.51       5834.06  0.000000E+00  0.000000E+00       2234.91  0.000000E+00       2936.18       3915.70  0.000000E+00       12191.0       51.4290       21885.7 -1.000000E+10 -1.000000E+10 -1.000000E+10 -1.000000E+10 -1.000000E+10  0.000000E+00  0.000000E+00  0.000000E+00  0.000000E+00  0.000000E+00  0.000000E+00  0.000000E+00  0.000000E+00  0.000000E+00  0.000000E+00  0.000000E+00  0.000000E+00       Funtag_D
      26  2.688945E+05  2.195083E+05       49386.2       70.4131      0.115947      0.654961      0.221656      0.234532    -0.0324302    -0.0375153    0.00688594       1.03202       Opttag_D
      26       50.7126       2.46498       1.00000  0.000000E+00       1.00000  0.000000E+00      0.823931       52.8257       1.00000       24.2775  0.000000E+00       2.32550  0.000000E+00       2.32550  0.000000E+00  0.000000E+00      -4.65099       52.1585       1.51622        1       Scatag_D
      26       1331.15       5804.13  0.000000E+00  0.000000E+00       2234.77  0.000000E+00       2889.65       3749.23  0.000000E+00       11958.0       55.8504       21363.4 -1.000000E+10 -1.000000E+10 -1.000000E+10 -1.000000E+10 -1.000000E+10  0.000000E+00  0.000000E+00  0.000000E+00  0.000000E+00  0.000000E+00  0.000000E+00  0.000000E+00  0.000000E+00  0.000000E+00  0.000000E+00  0.000000E+00  0.000000E+00       Funtag_D
      27  2.682074E+05  2.195232E+05       48684.2       60.8095      0.117254      0.671568      0.221696      0.234662    -0.0325133    -0.0378482    0.00677835       1.02196       Opttag_D
      27       50.7213       2.46701       1.00000  0.000000E+00       1.00000  0.000000E+00      0.823975       52.7584       1.00000       24.2784  0.000000E+00       2.32528  0.000000E+00       2.32528  0.000000E+00  0.000000E+00      -4.65056       52.1585       1.51622        1       Scatag_D
      27       1289.88       5691.55  0.000000E+00  0.000000E+00       2234.30  0.000000E+00       2867.33       3652.55  0.000000E+00       11837.3       60.6639       21050.6 -1.000000E+10 -1.000000E+10 -1.000000E+10 -1.000000E+10 -1.000000E+10  0.000000E+00  0.000000E+00  0.000000E+00  0.000000E+00  0.000000E+00  0.000000E+00  0.000000E+00  0.000000E+00  0.000000E+00  0.000000E+00  0.000000E+00  0.000000E+00       Funtag_D
      28  2.676703E+05  2.195143E+05       48155.9       43.5503      0.117783      0.671338      0.221689      0.234691    -0.0324642    -0.0379225    0.00668342       1.01168       Opttag_D
      28       50.7277       2.46700       1.00000  0.000000E+00       1.00000  0.000000E+00      0.823999       52.7340       1.00000       24.2818  0.000000E+00       2.32373  0.000000E+00       2.32373  0.000000E+00  0.000000E+00      -4.64745       52.1585       1.51622        1       Scatag_D
      28       1254.00       5577.63  0.000000E+00  0.000000E+00       2233.69  0.000000E+00       2856.39       3550.87  0.000000E+00       11785.7       62.1548       20835.5 -1.000000E+10 -1.000000E+10 -1.000000E+10 -1.000000E+10 -1.000000E+10  0.000000E+00  0.000000E+00  0.000000E+00  0.000000E+00  0.000000E+00  0.000000E+00  0.000000E+00  0.000000E+00  0.000000E+00  0.000000E+00  0.000000E+00  0.000000E+00       Funtag_D
      29  2.668207E+05  2.195212E+05       47299.5       42.1253      0.119865      0.672545      0.221721      0.234843    -0.0325026    -0.0383252    0.00659792       1.00074       Opttag_D
      29       50.7538       2.47104       1.00000  0.000000E+00       1.00000  0.000000E+00      0.824108       52.6590       1.00000       24.2867  0.000000E+00       2.32104  0.000000E+00       2.32104  0.000000E+00  0.000000E+00      -4.64207       52.1585       1.51622        1       Scatag_D
      29       1222.12       5457.67  0.000000E+00  0.000000E+00       2232.66  0.000000E+00       2828.47       3494.03  0.000000E+00       11611.1       62.2455       20391.2 -1.000000E+10 -1.000000E+10 -1.000000E+10 -1.000000E+10 -1.000000E+10  0.000000E+00  0.000000E+00  0.000000E+00  0.000000E+00  0.000000E+00  0.000000E+00  0.000000E+00  0.000000E+00  0.000000E+00  0.000000E+00  0.000000E+00  0.000000E+00       Funtag_D
      30  2.663935E+05  2.195965E+05       46797.0       72.9010      0.122343      0.682106      0.221866      0.235107    -0.0329220    -0.0390687    0.00660329       1.00777       Opttag_D
      30       50.7820       2.48126       1.00000  0.000000E+00       1.00000  0.000000E+00      0.824248       52.5804       1.00000       24.2833  0.000000E+00       2.32318  0.000000E+00       2.32318  0.000000E+00  0.000000E+00      -4.64637       52.1585       1.51622        1       Scatag_D
      30       1224.11       5534.52  0.000000E+00  0.000000E+00       2232.55  0.000000E+00       2797.18       3331.39  0.000000E+00       11435.1       56.7675       20185.4 -1.000000E+10 -1.000000E+10 -1.000000E+10 -1.000000E+10 -1.000000E+10  0.000000E+00  0.000000E+00  0.000000E+00  0.000000E+00  0.000000E+00  0.000000E+00  0.000000E+00  0.000000E+00  0.000000E+00  0.000000E+00  0.000000E+00  0.000000E+00       Funtag_D
      31  2.659305E+05  2.196341E+05       46296.4       43.7777      0.123352      0.682623      0.221934      0.235226    -0.0331314    -0.0394023    0.00653443      0.993143       Opttag_D
      31       50.7948       2.48631       1.00000  0.000000E+00       1.00000  0.000000E+00      0.824314       52.5522       1.00000       24.2799  0.000000E+00       2.32439  0.000000E+00       2.32439  0.000000E+00  0.000000E+00      -4.64878       52.1585       1.51622        1       Scatag_D
      31       1198.71       5375.07  0.000000E+00  0.000000E+00       2232.80  0.000000E+00       2778.10       3227.32  0.000000E+00       11362.7       53.4635       20068.2 -1.000000E+10 -1.000000E+10 -1.000000E+10 -1.000000E+10 -1.000000E+10  0.000000E+00  0.000000E+00  0.000000E+00  0.000000E+00  0.000000E+00  0.000000E+00  0.000000E+00  0.000000E+00  0.000000E+00  0.000000E+00  0.000000E+00  0.000000E+00       Funtag_D
      32  2.653279E+05  2.197501E+05       45577.8       33.5285      0.126071      0.702158      0.222142      0.235555    -0.0337777    -0.0403493    0.00648357      0.984594       Opttag_D
      32       50.8289       2.50117       1.00000  0.000000E+00       1.00000  0.000000E+00      0.824494       52.4801       1.00000       24.2679  0.000000E+00       2.32869  0.000000E+00       2.32869  0.000000E+00  0.000000E+00      -4.65737       52.1585       1.51622        1       Scatag_D
      32       1180.13       5282.94  0.000000E+00  0.000000E+00       2234.00  0.000000E+00       2725.92       3118.41  0.000000E+00       11178.8       47.5350       19810.1 -1.000000E+10 -1.000000E+10 -1.000000E+10 -1.000000E+10 -1.000000E+10  0.000000E+00  0.000000E+00  0.000000E+00  0.000000E+00  0.000000E+00  0.000000E+00  0.000000E+00  0.000000E+00  0.000000E+00  0.000000E+00  0.000000E+00  0.000000E+00       Funtag_D
      33  2.649230E+05  2.198463E+05       45076.6       42.3423      0.128385      0.719346      0.222310      0.235827    -0.0343136    -0.0411195    0.00644098      0.983652       Opttag_D
      33       50.8589       2.51371       1.00000  0.000000E+00       1.00000  0.000000E+00      0.824648       52.4194       1.00000       24.2582  0.000000E+00       2.33167  0.000000E+00       2.33167  0.000000E+00  0.000000E+00      -4.66334       52.1585       1.51622        1       Scatag_D
      33       1164.67       5272.84  0.000000E+00  0.000000E+00       2235.13  0.000000E+00       2680.62       3029.17  0.000000E+00       11029.6       46.9709       19617.7 -1.000000E+10 -1.000000E+10 -1.000000E+10 -1.000000E+10 -1.000000E+10  0.000000E+00  0.000000E+00  0.000000E+00  0.000000E+00  0.000000E+00  0.000000E+00  0.000000E+00  0.000000E+00  0.000000E+00  0.000000E+00  0.000000E+00  0.000000E+00       Funtag_D
      34  2.642480E+05  2.201111E+05       44136.9       54.5846      0.134345      0.746750      0.222762      0.236507    -0.0357887    -0.0431336    0.00642277      0.981942       Opttag_D
      34       50.9397       2.54823       1.00000  0.000000E+00       1.00000  0.000000E+00      0.825055       52.2782       1.00000       24.2341  0.000000E+00       2.33906  0.000000E+00       2.33906  0.000000E+00  0.000000E+00      -4.67811       52.1585       1.51622        1       Scatag_D
      34       1158.10       5254.52  0.000000E+00  0.000000E+00       2239.49  0.000000E+00       2572.44       2904.98  0.000000E+00       10688.8       48.8842       19269.7 -1.000000E+10 -1.000000E+10 -1.000000E+10 -1.000000E+10 -1.000000E+10  0.000000E+00  0.000000E+00  0.000000E+00  0.000000E+00  0.000000E+00  0.000000E+00  0.000000E+00  0.000000E+00  0.000000E+00  0.000000E+00  0.000000E+00  0.000000E+00       Funtag_D
      35  2.637619E+05  2.202207E+05       43541.2       60.0340      0.137126      0.741561      0.222937      0.236794    -0.0363990    -0.0439949    0.00638092      0.983207       Opttag_D
      35       50.9847       2.56445       1.00000  0.000000E+00       1.00000  0.000000E+00      0.825264       52.2211       1.00000       24.2156  0.000000E+00       2.34112  0.000000E+00       2.34112  0.000000E+00  0.000000E+00      -4.68223       52.1585       1.51622        1       Scatag_D
      35       1143.06       5268.07  0.000000E+00  0.000000E+00       2240.44  0.000000E+00       2520.16       2751.95  0.000000E+00       10526.1       50.8129       19040.6 -1.000000E+10 -1.000000E+10 -1.000000E+10 -1.000000E+10 -1.000000E+10  0.000000E+00  0.000000E+00  0.000000E+00  0.000000E+00  0.000000E+00  0.000000E+00  0.000000E+00  0.000000E+00  0.000000E+00  0.000000E+00  0.000000E+00  0.000000E+00       Funtag_D
      36  2.633670E+05  2.202046E+05       43162.4       36.9498      0.137202      0.728483      0.222904      0.236759    -0.0363095    -0.0439251    0.00629414      0.972858       Opttag_D
      36       50.9917       2.56409       1.00000  0.000000E+00       1.00000  0.000000E+00      0.825281       52.2194       1.00000       24.2086  0.000000E+00       2.34013  0.000000E+00       2.34013  0.000000E+00  0.000000E+00      -4.68025       52.1585       1.51622        1       Scatag_D
      36       1112.18       5157.75  0.000000E+00  0.000000E+00       2239.53  0.000000E+00       2510.27       2696.21  0.000000E+00       10502.4       51.4969       18892.5 -1.000000E+10 -1.000000E+10 -1.000000E+10 -1.000000E+10 -1.000000E+10  0.000000E+00  0.000000E+00  0.000000E+00  0.000000E+00  0.000000E+00  0.000000E+00  0.000000E+00  0.000000E+00  0.000000E+00  0.000000E+00  0.000000E+00  0.000000E+00       Funtag_D
      37  2.629984E+05  2.202088E+05       42789.6       29.9614      0.137981      0.709162      0.222892      0.236797    -0.0363327    -0.0440733    0.00624904      0.968049       Opttag_D
      37       51.0145       2.56794       1.00000  0.000000E+00       1.00000  0.000000E+00      0.825360       52.2063       1.00000       24.1931  0.000000E+00       2.33858  0.000000E+00       2.33858  0.000000E+00  0.000000E+00      -4.67715       52.1585       1.51622        1       Scatag_D
      37       1096.29       5106.88  0.000000E+00  0.000000E+00       2238.10  0.000000E+00       2489.35       2682.74  0.000000E+00       10437.8       50.7542       18687.8 -1.000000E+10 -1.000000E+10 -1.000000E+10 -1.000000E+10 -1.000000E+10  0.000000E+00  0.000000E+00  0.000000E+00  0.000000E+00  0.000000E+00  0.000000E+00  0.000000E+00  0.000000E+00  0.000000E+00  0.000000E+00  0.000000E+00  0.000000E+00       Funtag_D
      38  2.627120E+05  2.202738E+05       42438.2       37.7886      0.139958      0.695512      0.222977      0.237000    -0.0366949    -0.0447083    0.00625674      0.967480       Opttag_D
      38       51.0565       2.58006       1.00000  0.000000E+00       1.00000  0.000000E+00      0.825530       52.1725       1.00000       24.1728  0.000000E+00       2.33765  0.000000E+00       2.33765  0.000000E+00  0.000000E+00      -4.67529       52.1585       1.51622        1       Scatag_D
      38       1099.00       5100.88  0.000000E+00  0.000000E+00       2237.85  0.000000E+00       2455.82       2651.39  0.000000E+00       10327.3       48.4443       18517.5 -1.000000E+10 -1.000000E+10 -1.000000E+10 -1.000000E+10 -1.000000E+10  0.000000E+00  0.000000E+00  0.000000E+00  0.000000E+00  0.000000E+00  0.000000E+00  0.000000E+00  0.000000E+00  0.000000E+00  0.000000E+00  0.000000E+00  0.000000E+00       Funtag_D
      39  2.623443E+05  2.203792E+05       41965.2       30.2463      0.142549      0.688141      0.223129      0.237315    -0.0372818    -0.0456570    0.00621284      0.962388       Opttag_D
      39       51.1080       2.59724       1.00000  0.000000E+00       1.00000  0.000000E+00      0.825746       52.1296       1.00000       24.1518  0.000000E+00       2.33710  0.000000E+00       2.33710  0.000000E+00  0.000000E+00      -4.67420       52.1585       1.51622        1       Scatag_D
      39       1083.63       5047.33  0.000000E+00  0.000000E+00       2237.83  0.000000E+00       2416.95       2562.06  0.000000E+00       10203.6       45.4382       18368.3 -1.000000E+10 -1.000000E+10 -1.000000E+10 -1.000000E+10 -1.000000E+10  0.000000E+00  0.000000E+00  0.000000E+00  0.000000E+00  0.000000E+00  0.000000E+00  0.000000E+00  0.000000E+00  0.000000E+00  0.000000E+00  0.000000E+00  0.000000E+00       Funtag_D
      40  2.618707E+05  2.206174E+05       41253.3       45.9151      0.147794      0.686344      0.223483      0.238013    -0.0386090    -0.0477109    0.00620561      0.962255       Opttag_D
      40       51.2127       2.63469       1.00000  0.000000E+00       1.00000  0.000000E+00      0.826181       52.0529       1.00000       24.1131  0.000000E+00       2.33619  0.000000E+00       2.33619  0.000000E+00  0.000000E+00      -4.67239       52.1585       1.51622        1       Scatag_D
      40       1081.11       5045.93  0.000000E+00  0.000000E+00       2238.51  0.000000E+00       2343.08       2449.52  0.000000E+00       9982.13       41.4757       18071.6 -1.000000E+10 -1.000000E+10 -1.000000E+10 -1.000000E+10 -1.000000E+10  0.000000E+00  0.000000E+00  0.000000E+00  0.000000E+00  0.000000E+00  0.000000E+00  0.000000E+00  0.000000E+00  0.000000E+00  0.000000E+00  0.000000E+00  0.000000E+00       Funtag_D
      41  2.615359E+05  2.208080E+05       40727.9       42.4339      0.151694      0.710931      0.223775      0.238570    -0.0396707    -0.0493332    0.00619645      0.958463       Opttag_D
      41       51.2912       2.66395       1.00000  0.000000E+00       1.00000  0.000000E+00      0.826496       51.9994       1.00000       24.0905  0.000000E+00       2.33572  0.000000E+00       2.33572  0.000000E+00  0.000000E+00      -4.67145       52.1585       1.51622        1       Scatag_D
      41       1077.92       5006.24  0.000000E+00  0.000000E+00       2239.48  0.000000E+00       2292.49       2414.52  0.000000E+00       9834.65       40.7370       17821.8 -1.000000E+10 -1.000000E+10 -1.000000E+10 -1.000000E+10 -1.000000E+10  0.000000E+00  0.000000E+00  0.000000E+00  0.000000E+00  0.000000E+00  0.000000E+00  0.000000E+00  0.000000E+00  0.000000E+00  0.000000E+00  0.000000E+00  0.000000E+00       Funtag_D
      42  2.612385E+05  2.208092E+05       40429.3       30.5205      0.151817      0.721285      0.223776      0.238602    -0.0396776    -0.0494090    0.00611045      0.953761       Opttag_D
      42       51.2973       2.66503       1.00000  0.000000E+00       1.00000  0.000000E+00      0.826505       51.9980       1.00000       24.0872  0.000000E+00       2.33522  0.000000E+00       2.33522  0.000000E+00  0.000000E+00      -4.67045       52.1585       1.51622        1       Scatag_D
      42       1048.21       4957.25  0.000000E+00  0.000000E+00       2238.68  0.000000E+00       2285.65       2367.60  0.000000E+00       9817.53       42.2097       17672.1 -1.000000E+10 -1.000000E+10 -1.000000E+10 -1.000000E+10 -1.000000E+10  0.000000E+00  0.000000E+00  0.000000E+00  0.000000E+00  0.000000E+00  0.000000E+00  0.000000E+00  0.000000E+00  0.000000E+00  0.000000E+00  0.000000E+00  0.000000E+00       Funtag_D
      43  2.607355E+05  2.208724E+05       39863.0       26.2525      0.153593      0.777966      0.223862      0.238875    -0.0400296    -0.0501441    0.00604881      0.949633       Opttag_D
      43       51.3454       2.67813       1.00000  0.000000E+00       1.00000  0.000000E+00      0.826640       51.9748       1.00000       24.0738  0.000000E+00       2.33260  0.000000E+00       2.33260  0.000000E+00  0.000000E+00      -4.66519       52.1585       1.51622        1       Scatag_D
      43       1027.16       4914.43  0.000000E+00  0.000000E+00       2237.53  0.000000E+00       2262.79       2325.36  0.000000E+00       9723.65       46.5212       17325.6 -1.000000E+10 -1.000000E+10 -1.000000E+10 -1.000000E+10 -1.000000E+10  0.000000E+00  0.000000E+00  0.000000E+00  0.000000E+00  0.000000E+00  0.000000E+00  0.000000E+00  0.000000E+00  0.000000E+00  0.000000E+00  0.000000E+00  0.000000E+00       Funtag_D
      44  2.604477E+05  2.210379E+05       39409.8       51.3898      0.157421      0.857091      0.224116      0.239434    -0.0409513    -0.0516519    0.00606805      0.956745       Opttag_D
      44       51.4338       2.70674       1.00000  0.000000E+00       1.00000  0.000000E+00      0.826941       51.9261       1.00000       24.0601  0.000000E+00       2.32929  0.000000E+00       2.32929  0.000000E+00  0.000000E+00      -4.65858       52.1585       1.51622        1       Scatag_D
      44       1033.71       4988.31  0.000000E+00  0.000000E+00       2238.01  0.000000E+00       2230.14       2243.15  0.000000E+00       9578.83       45.5903       17052.1 -1.000000E+10 -1.000000E+10 -1.000000E+10 -1.000000E+10 -1.000000E+10  0.000000E+00  0.000000E+00  0.000000E+00  0.000000E+00  0.000000E+00  0.000000E+00  0.000000E+00  0.000000E+00  0.000000E+00  0.000000E+00  0.000000E+00  0.000000E+00       Funtag_D
      45  2.601003E+05  2.211129E+05       38987.4       32.7621      0.159354      0.891306      0.224226      0.239718    -0.0413693    -0.0523910    0.00599447      0.947356       Opttag_D
      45       51.4826       2.72100       1.00000  0.000000E+00       1.00000  0.000000E+00      0.827092       51.9011       1.00000       24.0570  0.000000E+00       2.32675  0.000000E+00       2.32675  0.000000E+00  0.000000E+00      -4.65351       52.1585       1.51622        1       Scatag_D
      45       1008.79       4890.89  0.000000E+00  0.000000E+00       2238.24  0.000000E+00       2214.88       2207.95  0.000000E+00       9503.43       44.6538       16878.6 -1.000000E+10 -1.000000E+10 -1.000000E+10 -1.000000E+10 -1.000000E+10  0.000000E+00  0.000000E+00  0.000000E+00  0.000000E+00  0.000000E+00  0.000000E+00  0.000000E+00  0.000000E+00  0.000000E+00  0.000000E+00  0.000000E+00  0.000000E+00       Funtag_D
      46  2.597712E+05  2.211623E+05       38608.9       26.2387      0.160865      0.900360      0.224287      0.239913    -0.0416444    -0.0529071    0.00594131      0.942519       Opttag_D
      46       51.5286       2.73254       1.00000  0.000000E+00       1.00000  0.000000E+00      0.827220       51.8873       1.00000       24.0523  0.000000E+00       2.32399  0.000000E+00       2.32399  0.000000E+00  0.000000E+00      -4.64798       52.1585       1.51622        1       Scatag_D
      46       990.978       4841.06  0.000000E+00  0.000000E+00       2238.07  0.000000E+00       2196.70       2203.13  0.000000E+00       9433.75       42.6656       16662.5 -1.000000E+10 -1.000000E+10 -1.000000E+10 -1.000000E+10 -1.000000E+10  0.000000E+00  0.000000E+00  0.000000E+00  0.000000E+00  0.000000E+00  0.000000E+00  0.000000E+00  0.000000E+00  0.000000E+00  0.000000E+00  0.000000E+00  0.000000E+00       Funtag_D
      47  2.595930E+05  2.211589E+05       38434.1       28.0045      0.160957      0.884258      0.224267      0.239909    -0.0416256    -0.0529191    0.00591528      0.941533       Opttag_D
      47       51.5409       2.73427       1.00000  0.000000E+00       1.00000  0.000000E+00      0.827247       51.8962       1.00000       24.0438  0.000000E+00       2.32315  0.000000E+00       2.32315  0.000000E+00  0.000000E+00      -4.64631       52.1585       1.51622        1       Scatag_D
      47       982.316       4830.95  0.000000E+00  0.000000E+00       2237.22  0.000000E+00       2188.66       2173.87  0.000000E+00       9414.62       42.1201       16564.4 -1.000000E+10 -1.000000E+10 -1.000000E+10 -1.000000E+10 -1.000000E+10  0.000000E+00  0.000000E+00  0.000000E+00  0.000000E+00  0.000000E+00  0.000000E+00  0.000000E+00  0.000000E+00  0.000000E+00  0.000000E+00  0.000000E+00  0.000000E+00       Funtag_D
      48  2.593924E+05  2.211577E+05       38234.7       20.8493      0.161100      0.881008      0.224246      0.239903    -0.0416187    -0.0529474    0.00587476      0.938084       Opttag_D
      48       51.5581       2.73713       1.00000  0.000000E+00       1.00000  0.000000E+00      0.827286       51.9109       1.00000       24.0301  0.000000E+00       2.32206  0.000000E+00       2.32206  0.000000E+00  0.000000E+00      -4.64411       52.1585       1.51622        1       Scatag_D
      48       968.904       4795.62  0.000000E+00  0.000000E+00       2236.26  0.000000E+00       2179.38       2136.67  0.000000E+00       9389.47       42.2153       16486.2 -1.000000E+10 -1.000000E+10 -1.000000E+10 -1.000000E+10 -1.000000E+10  0.000000E+00  0.000000E+00  0.000000E+00  0.000000E+00  0.000000E+00  0.000000E+00  0.000000E+00  0.000000E+00  0.000000E+00  0.000000E+00  0.000000E+00  0.000000E+00       Funtag_D
      49  2.592259E+05  2.211959E+05       38030.0       39.3405      0.162327      0.903459      0.224264      0.240030    -0.0418317    -0.0533475    0.00585899      0.939292       Opttag_D
      49       51.6139       2.75015       1.00000  0.000000E+00       1.00000  0.000000E+00      0.827436       51.9357       1.00000       23.9978  0.000000E+00       2.31822  0.000000E+00       2.31822  0.000000E+00  0.000000E+00      -4.63643       52.1585       1.51622        1       Scatag_D
      49       963.710       4807.98  0.000000E+00  0.000000E+00       2234.52  0.000000E+00       2160.07       2100.72  0.000000E+00       9312.19       43.3242       16407.5 -1.000000E+10 -1.000000E+10 -1.000000E+10 -1.000000E+10 -1.000000E+10  0.000000E+00  0.000000E+00  0.000000E+00  0.000000E+00  0.000000E+00  0.000000E+00  0.000000E+00  0.000000E+00  0.000000E+00  0.000000E+00  0.000000E+00  0.000000E+00       Funtag_D
      50  2.590543E+05  2.212222E+05       37832.1       19.5557      0.163208      0.909495      0.224289      0.240134    -0.0419779    -0.0536376    0.00580616      0.932185       Opttag_D
      50       51.6468       2.75802       1.00000  0.000000E+00       1.00000  0.000000E+00      0.827527       51.9415       1.00000       23.9855  0.000000E+00       2.31538  0.000000E+00       2.31538  0.000000E+00  0.000000E+00      -4.63075       52.1585       1.51622        1       Scatag_D
      50       946.408       4735.49  0.000000E+00  0.000000E+00       2234.03  0.000000E+00       2152.06       2083.22  0.000000E+00       9268.73       43.5765       16368.6 -1.000000E+10 -1.000000E+10 -1.000000E+10 -1.000000E+10 -1.000000E+10  0.000000E+00  0.000000E+00  0.000000E+00  0.000000E+00  0.000000E+00  0.000000E+00  0.000000E+00  0.000000E+00  0.000000E+00  0.000000E+00  0.000000E+00  0.000000E+00       Funtag_D
      51  2.589385E+05  2.212461E+05       37692.4       19.1577      0.164072      0.905927      0.224317      0.240231    -0.0421113    -0.0539045    0.00578698      0.930425       Opttag_D
      51       51.6762       2.76507       1.00000  0.000000E+00       1.00000  0.000000E+00      0.827608       51.9437       1.00000       23.9771  0.000000E+00       2.31242  0.000000E+00       2.31242  0.000000E+00  0.000000E+00      -4.62484       52.1585       1.51622        1       Scatag_D
      51       940.165       4717.63  0.000000E+00  0.000000E+00       2233.69  0.000000E+00       2146.25       2068.37  0.000000E+00       9229.77       43.5132       16313.0 -1.000000E+10 -1.000000E+10 -1.000000E+10 -1.000000E+10 -1.000000E+10  0.000000E+00  0.000000E+00  0.000000E+00  0.000000E+00  0.000000E+00  0.000000E+00  0.000000E+00  0.000000E+00  0.000000E+00  0.000000E+00  0.000000E+00  0.000000E+00       Funtag_D
      52  2.586912E+05  2.213160E+05       37375.2       25.2899      0.166753      0.881616      0.224391      0.240516    -0.0425006    -0.0546953    0.00578653      0.927018       Opttag_D
      52       51.7711       2.78731       1.00000  0.000000E+00       1.00000  0.000000E+00      0.827865       51.9578       1.00000       23.9500  0.000000E+00       2.30232  0.000000E+00       2.30232  0.000000E+00  0.000000E+00      -4.60463       52.1585       1.51622        1       Scatag_D
      52       940.019       4683.14  0.000000E+00  0.000000E+00       2232.80  0.000000E+00       2132.56       2062.97  0.000000E+00       9113.58       42.7460       16167.3 -1.000000E+10 -1.000000E+10 -1.000000E+10 -1.000000E+10 -1.000000E+10  0.000000E+00  0.000000E+00  0.000000E+00  0.000000E+00  0.000000E+00  0.000000E+00  0.000000E+00  0.000000E+00  0.000000E+00  0.000000E+00  0.000000E+00  0.000000E+00       Funtag_D
      53  2.586611E+05  2.214174E+05       37243.7       68.1068      0.170757      0.885570      0.224500      0.240893    -0.0430654    -0.0557897    0.00585306      0.938594       Opttag_D
      53       51.9192       2.82150       1.00000  0.000000E+00       1.00000  0.000000E+00      0.828258       51.9903       1.00000       23.9149  0.000000E+00       2.28653  0.000000E+00       2.28653  0.000000E+00  0.000000E+00      -4.57307       52.1585       1.51622        1       Scatag_D
      53       961.758       4800.83  0.000000E+00  0.000000E+00       2231.92  0.000000E+00       2113.15       2093.67  0.000000E+00       8956.31       41.6542       16044.5 -1.000000E+10 -1.000000E+10 -1.000000E+10 -1.000000E+10 -1.000000E+10  0.000000E+00  0.000000E+00  0.000000E+00  0.000000E+00  0.000000E+00  0.000000E+00  0.000000E+00  0.000000E+00  0.000000E+00  0.000000E+00  0.000000E+00  0.000000E+00       Funtag_D
      54  2.583713E+05  2.214065E+05       36964.7       22.4128      0.170498      0.882609      0.224478      0.240860    -0.0430051    -0.0557103    0.00574190      0.921827       Opttag_D
      54       51.9119       2.81932       1.00000  0.000000E+00       1.00000  0.000000E+00      0.828236       51.9918       1.00000       23.9143  0.000000E+00       2.28703  0.000000E+00       2.28703  0.000000E+00  0.000000E+00      -4.57407       52.1585       1.51622        1       Scatag_D
      54       925.573       4630.84  0.000000E+00  0.000000E+00       2231.75  0.000000E+00       2113.37       2036.17  0.000000E+00       8962.23       41.5146       16023.3 -1.000000E+10 -1.000000E+10 -1.000000E+10 -1.000000E+10 -1.000000E+10  0.000000E+00  0.000000E+00  0.000000E+00  0.000000E+00  0.000000E+00  0.000000E+00  0.000000E+00  0.000000E+00  0.000000E+00  0.000000E+00  0.000000E+00  0.000000E+00       Funtag_D
      55  2.582673E+05  2.214092E+05       36858.0       12.8846      0.170869      0.882868      0.224473      0.240878    -0.0430200    -0.0557772    0.00570685      0.919907       Opttag_D
      55       51.9304       2.82273       1.00000  0.000000E+00       1.00000  0.000000E+00      0.828279       52.0016       1.00000       23.9078  0.000000E+00       2.28481  0.000000E+00       2.28481  0.000000E+00  0.000000E+00      -4.56961       52.1585       1.51622        1       Scatag_D
      55       914.311       4611.57  0.000000E+00  0.000000E+00       2231.27  0.000000E+00       2108.50       2019.17  0.000000E+00       8942.86       41.3570       15989.0 -1.000000E+10 -1.000000E+10 -1.000000E+10 -1.000000E+10 -1.000000E+10  0.000000E+00  0.000000E+00  0.000000E+00  0.000000E+00  0.000000E+00  0.000000E+00  0.000000E+00  0.000000E+00  0.000000E+00  0.000000E+00  0.000000E+00  0.000000E+00       Funtag_D
      56  2.581528E+05  2.214341E+05       36718.8       17.0206      0.172139      0.886376      0.224487      0.240976    -0.0431583    -0.0560847    0.00568015      0.919463       Opttag_D
      56       51.9842       2.83420       1.00000  0.000000E+00       1.00000  0.000000E+00      0.828414       52.0239       1.00000       23.8926  0.000000E+00       2.27895  0.000000E+00       2.27895  0.000000E+00  0.000000E+00      -4.55791       52.1585       1.51622        1       Scatag_D
      56       905.774       4607.13  0.000000E+00  0.000000E+00       2230.50  0.000000E+00       2095.94       2016.47  0.000000E+00       8890.39       41.3956       15931.2 -1.000000E+10 -1.000000E+10 -1.000000E+10 -1.000000E+10 -1.000000E+10  0.000000E+00  0.000000E+00  0.000000E+00  0.000000E+00  0.000000E+00  0.000000E+00  0.000000E+00  0.000000E+00  0.000000E+00  0.000000E+00  0.000000E+00  0.000000E+00       Funtag_D
      57  2.579910E+05  2.214729E+05       36518.1       23.8758      0.174002      0.888196      0.224518      0.241122    -0.0433746    -0.0565461    0.00565441      0.920147       Opttag_D
      57       52.0620       2.85118       1.00000  0.000000E+00       1.00000  0.000000E+00      0.828605       52.0587       1.00000       23.8704  0.000000E+00       2.27080  0.000000E+00       2.27080  0.000000E+00  0.000000E+00      -4.54160       52.1585       1.51622        1       Scatag_D
      57       897.584       4613.98  0.000000E+00  0.000000E+00       2229.10  0.000000E+00       2074.12       2014.55  0.000000E+00       8816.64       41.9791       15830.2 -1.000000E+10 -1.000000E+10 -1.000000E+10 -1.000000E+10 -1.000000E+10  0.000000E+00  0.000000E+00  0.000000E+00  0.000000E+00  0.000000E+00  0.000000E+00  0.000000E+00  0.000000E+00  0.000000E+00  0.000000E+00  0.000000E+00  0.000000E+00       Funtag_D
      58  2.578258E+05  2.215963E+05       36229.5       39.8863      0.178964      0.891324      0.224641      0.241563    -0.0440623    -0.0578878    0.00569475      0.923095       Opttag_D
      58       52.2584       2.89666       1.00000  0.000000E+00       1.00000  0.000000E+00      0.829102       52.1439       1.00000       23.8250  0.000000E+00       2.25071  0.000000E+00       2.25071  0.000000E+00  0.000000E+00      -4.50142       52.1585       1.51622        1       Scatag_D
      58       910.437       4643.59  0.000000E+00  0.000000E+00       2227.42  0.000000E+00       2029.40       2066.24  0.000000E+00       8660.38       43.1329       15648.9 -1.000000E+10 -1.000000E+10 -1.000000E+10 -1.000000E+10 -1.000000E+10  0.000000E+00  0.000000E+00  0.000000E+00  0.000000E+00  0.000000E+00  0.000000E+00  0.000000E+00  0.000000E+00  0.000000E+00  0.000000E+00  0.000000E+00  0.000000E+00       Funtag_D
      59  2.576055E+05  2.216132E+05       35992.3       18.8850      0.179918      0.888756      0.224657      0.241638    -0.0441562    -0.0580921    0.00564326      0.919718       Opttag_D
      59       52.2965       2.90500       1.00000  0.000000E+00       1.00000  0.000000E+00      0.829192       52.1622       1.00000       23.8160  0.000000E+00       2.24631  0.000000E+00       2.24631  0.000000E+00  0.000000E+00      -4.49262       52.1585       1.51622        1       Scatag_D
      59       894.045       4609.68  0.000000E+00  0.000000E+00       2226.45  0.000000E+00       2020.20       2017.69  0.000000E+00       8629.12       43.0421       15552.1 -1.000000E+10 -1.000000E+10 -1.000000E+10 -1.000000E+10 -1.000000E+10  0.000000E+00  0.000000E+00  0.000000E+00  0.000000E+00  0.000000E+00  0.000000E+00  0.000000E+00  0.000000E+00  0.000000E+00  0.000000E+00  0.000000E+00  0.000000E+00       Funtag_D
      60  2.574389E+05  2.216207E+05       35818.1       20.9506      0.180734      0.899201      0.224659      0.241698    -0.0441982    -0.0582534    0.00561134      0.919703       Opttag_D
      60       52.3297       2.91162       1.00000  0.000000E+00       1.00000  0.000000E+00      0.829267       52.1781       1.00000       23.8078  0.000000E+00       2.24189  0.000000E+00       2.24189  0.000000E+00  0.000000E+00      -4.48379       52.1585       1.51622        1       Scatag_D
      60       883.962       4609.53  0.000000E+00  0.000000E+00       2225.30  0.000000E+00       2012.37       2012.49  0.000000E+00       8600.44       42.7213       15431.3 -1.000000E+10 -1.000000E+10 -1.000000E+10 -1.000000E+10 -1.000000E+10  0.000000E+00  0.000000E+00  0.000000E+00  0.000000E+00  0.000000E+00  0.000000E+00  0.000000E+00  0.000000E+00  0.000000E+00  0.000000E+00  0.000000E+00  0.000000E+00       Funtag_D
      61  2.572345E+05  2.216640E+05       35570.6       25.0031      0.183217      0.937377      0.224693      0.241889    -0.0444389    -0.0588103    0.00560085      0.918707       Opttag_D
      61       52.4264       2.93288       1.00000  0.000000E+00       1.00000  0.000000E+00      0.829501       52.2235       1.00000       23.7879  0.000000E+00       2.23013  0.000000E+00       2.23013  0.000000E+00  0.000000E+00      -4.46026       52.1585       1.51622        1       Scatag_D
      61       880.660       4599.55  0.000000E+00  0.000000E+00       2223.40  0.000000E+00       1997.67       2038.30  0.000000E+00       8532.65       42.2877       15256.1 -1.000000E+10 -1.000000E+10 -1.000000E+10 -1.000000E+10 -1.000000E+10  0.000000E+00  0.000000E+00  0.000000E+00  0.000000E+00  0.000000E+00  0.000000E+00  0.000000E+00  0.000000E+00  0.000000E+00  0.000000E+00  0.000000E+00  0.000000E+00       Funtag_D
      62  2.571036E+05  2.217006E+05       35403.0       32.5723      0.185291      0.969560      0.224723      0.242045    -0.0446431    -0.0592660    0.00559032      0.917877       Opttag_D
      62       52.5064       2.95053       1.00000  0.000000E+00       1.00000  0.000000E+00      0.829696       52.2583       1.00000       23.7754  0.000000E+00       2.21996  0.000000E+00       2.21996  0.000000E+00  0.000000E+00      -4.43993       52.1585       1.51622        2       Scatag_D
      62       877.351       4591.25  0.000000E+00  0.000000E+00       2221.45  0.000000E+00       1988.16       2041.28  0.000000E+00       8482.33       41.9355       15159.3 -1.000000E+10 -1.000000E+10 -1.000000E+10 -1.000000E+10 -1.000000E+10  0.000000E+00  0.000000E+00  0.000000E+00  0.000000E+00  0.000000E+00  0.000000E+00  0.000000E+00  0.000000E+00  0.000000E+00  0.000000E+00  0.000000E+00  0.000000E+00       Funtag_D
      63  2.569406E+05  2.217054E+05       35235.2       19.2598      0.186117      0.984724      0.224711      0.242089    -0.0446697    -0.0593927    0.00554431      0.912022       Opttag_D
      63       52.5402       2.95704       1.00000  0.000000E+00       1.00000  0.000000E+00      0.829775       52.2745       1.00000       23.7685  0.000000E+00       2.21531  0.000000E+00       2.21531  0.000000E+00  0.000000E+00      -4.43061       52.1585       1.51622        1       Scatag_D
      63       862.970       4532.85  0.000000E+00  0.000000E+00       2219.92  0.000000E+00       1985.61       2025.21  0.000000E+00       8461.11       41.4887       15106.0 -1.000000E+10 -1.000000E+10 -1.000000E+10 -1.000000E+10 -1.000000E+10  0.000000E+00  0.000000E+00  0.000000E+00  0.000000E+00  0.000000E+00  0.000000E+00  0.000000E+00  0.000000E+00  0.000000E+00  0.000000E+00  0.000000E+00  0.000000E+00       Funtag_D
      64  2.567708E+05  2.216959E+05       35074.9       17.4814      0.186656      0.998660      0.224675      0.242091    -0.0446171    -0.0594209    0.00550618      0.905898       Opttag_D
      64       52.5663       2.96099       1.00000  0.000000E+00       1.00000  0.000000E+00      0.829829       52.2916       1.00000       23.7588  0.000000E+00       2.21121  0.000000E+00       2.21121  0.000000E+00  0.000000E+00      -4.42242       52.1585       1.51622        1       Scatag_D
      64       851.139       4472.19  0.000000E+00  0.000000E+00       2217.17  0.000000E+00       1984.48       2029.72  0.000000E+00       8443.18       41.2339       15035.8 -1.000000E+10 -1.000000E+10 -1.000000E+10 -1.000000E+10 -1.000000E+10  0.000000E+00  0.000000E+00  0.000000E+00  0.000000E+00  0.000000E+00  0.000000E+00  0.000000E+00  0.000000E+00  0.000000E+00  0.000000E+00  0.000000E+00  0.000000E+00       Funtag_D
      65  2.566660E+05  2.216986E+05       34967.4       23.1453      0.187515       1.01691      0.224660      0.242127    -0.0446320    -0.0595294    0.00550478      0.900837       Opttag_D
      65       52.6038       2.96806       1.00000  0.000000E+00       1.00000  0.000000E+00      0.829909       52.3135       1.00000       23.7482  0.000000E+00       2.20642  0.000000E+00       2.20642  0.000000E+00  0.000000E+00      -4.41284       52.1585       1.51622        1       Scatag_D
      65       850.708       4422.36  0.000000E+00  0.000000E+00       2213.91  0.000000E+00       1980.65       2049.80  0.000000E+00       8418.41       41.2973       14990.3 -1.000000E+10 -1.000000E+10 -1.000000E+10 -1.000000E+10 -1.000000E+10  0.000000E+00  0.000000E+00  0.000000E+00  0.000000E+00  0.000000E+00  0.000000E+00  0.000000E+00  0.000000E+00  0.000000E+00  0.000000E+00  0.000000E+00  0.000000E+00       Funtag_D
      66  2.565495E+05  2.217006E+05       34848.9       14.9574      0.187945       1.02719      0.224653      0.242154    -0.0446433    -0.0596128    0.00550699      0.897705       Opttag_D
      66       52.6241       2.97197       1.00000  0.000000E+00       1.00000  0.000000E+00      0.829947       52.3288       1.00000       23.7399  0.000000E+00       2.20420  0.000000E+00       2.20420  0.000000E+00  0.000000E+00      -4.40840       52.1585       1.51622        1       Scatag_D
      66       851.391       4391.66  0.000000E+00  0.000000E+00       2211.58  0.000000E+00       1976.58       2021.32  0.000000E+00       8402.80       41.4383       14952.1 -1.000000E+10 -1.000000E+10 -1.000000E+10 -1.000000E+10 -1.000000E+10  0.000000E+00  0.000000E+00  0.000000E+00  0.000000E+00  0.000000E+00  0.000000E+00  0.000000E+00  0.000000E+00  0.000000E+00  0.000000E+00  0.000000E+00  0.000000E+00       Funtag_D
      67  2.564359E+05  2.217041E+05       34731.9       13.7384      0.188413       1.03810      0.224646      0.242192    -0.0446624    -0.0597320    0.00553123      0.895429       Opttag_D
      67       52.6471       2.97653       1.00000  0.000000E+00       1.00000  0.000000E+00      0.829983       52.3483       1.00000       23.7301  0.000000E+00       2.20171  0.000000E+00       2.20171  0.000000E+00  0.000000E+00      -4.40343       52.1585       1.51622        1       Scatag_D
      67       858.901       4369.41  0.000000E+00  0.000000E+00       2209.06  0.000000E+00       1969.63       2008.19  0.000000E+00       8383.79       41.6552       14891.2 -1.000000E+10 -1.000000E+10 -1.000000E+10 -1.000000E+10 -1.000000E+10  0.000000E+00  0.000000E+00  0.000000E+00  0.000000E+00  0.000000E+00  0.000000E+00  0.000000E+00  0.000000E+00  0.000000E+00  0.000000E+00  0.000000E+00  0.000000E+00       Funtag_D
      68  2.563617E+05  2.217063E+05       34655.4       19.9961      0.189014       1.05015      0.224638      0.242245    -0.0446750    -0.0598545    0.00555663      0.894312       Opttag_D
      68       52.6746       2.98162       1.00000  0.000000E+00       1.00000  0.000000E+00      0.830024       52.3660       1.00000       23.7229  0.000000E+00       2.19838  0.000000E+00       2.19838  0.000000E+00  0.000000E+00      -4.39677       52.1585       1.51622        1       Scatag_D
      68       866.808       4358.52  0.000000E+00  0.000000E+00       2207.11  0.000000E+00       1965.36       2018.36  0.000000E+00       8364.30       41.4593       14833.5 -1.000000E+10 -1.000000E+10 -1.000000E+10 -1.000000E+10 -1.000000E+10  0.000000E+00  0.000000E+00  0.000000E+00  0.000000E+00  0.000000E+00  0.000000E+00  0.000000E+00  0.000000E+00  0.000000E+00  0.000000E+00  0.000000E+00  0.000000E+00       Funtag_D
      69  2.562767E+05  2.217035E+05       34573.2       14.5409      0.189458       1.05872      0.224624      0.242282    -0.0446592    -0.0599313    0.00555250      0.893153       Opttag_D
      69       52.6950       2.98498       1.00000  0.000000E+00       1.00000  0.000000E+00      0.830051       52.3783       1.00000       23.7191  0.000000E+00       2.19542  0.000000E+00       2.19542  0.000000E+00  0.000000E+00      -4.39084       52.1585       1.51622        1       Scatag_D
      69       865.522       4347.23  0.000000E+00  0.000000E+00       2206.05  0.000000E+00       1964.40       2006.35  0.000000E+00       8351.35       41.1339       14791.1 -1.000000E+10 -1.000000E+10 -1.000000E+10 -1.000000E+10 -1.000000E+10  0.000000E+00  0.000000E+00  0.000000E+00  0.000000E+00  0.000000E+00  0.000000E+00  0.000000E+00  0.000000E+00  0.000000E+00  0.000000E+00  0.000000E+00  0.000000E+00       Funtag_D
      70  2.561218E+05  2.216987E+05       34423.1       16.1485      0.190915       1.08504      0.224587      0.242390    -0.0446326    -0.0601633    0.00554328      0.891180       Opttag_D
      70       52.7592       2.99610       1.00000  0.000000E+00       1.00000  0.000000E+00      0.830144       52.4174       1.00000       23.7072  0.000000E+00       2.18604  0.000000E+00       2.18604  0.000000E+00  0.000000E+00      -4.37208       52.1585       1.51622        1       Scatag_D
      70       862.648       4328.05  0.000000E+00  0.000000E+00       2203.63  0.000000E+00       1966.58       2016.80  0.000000E+00       8316.90       40.3708       14688.1 -1.000000E+10 -1.000000E+10 -1.000000E+10 -1.000000E+10 -1.000000E+10  0.000000E+00  0.000000E+00  0.000000E+00  0.000000E+00  0.000000E+00  0.000000E+00  0.000000E+00  0.000000E+00  0.000000E+00  0.000000E+00  0.000000E+00  0.000000E+00       Funtag_D
      71  2.560231E+05  2.217003E+05       34322.8       23.8609      0.192246       1.10717      0.224569      0.242494    -0.0446412    -0.0604046    0.00552346      0.890580       Opttag_D
      71       52.8156       3.00636       1.00000  0.000000E+00       1.00000  0.000000E+00      0.830227       52.4502       1.00000       23.6980  0.000000E+00       2.17879  0.000000E+00       2.17879  0.000000E+00  0.000000E+00      -4.35758       52.1585       1.51622        1       Scatag_D
      71       856.490       4322.22  0.000000E+00  0.000000E+00       2202.00  0.000000E+00       1970.04       2040.79  0.000000E+00       8290.35       40.0687       14600.9 -1.000000E+10 -1.000000E+10 -1.000000E+10 -1.000000E+10 -1.000000E+10  0.000000E+00  0.000000E+00  0.000000E+00  0.000000E+00  0.000000E+00  0.000000E+00  0.000000E+00  0.000000E+00  0.000000E+00  0.000000E+00  0.000000E+00  0.000000E+00       Funtag_D
      72  2.559069E+05  2.217057E+05       34201.2       18.2532      0.193226       1.12237      0.224562      0.242564    -0.0446717    -0.0605791    0.00548794      0.889366       Opttag_D
      72       52.8562       3.01424       1.00000  0.000000E+00       1.00000  0.000000E+00      0.830290       52.4749       1.00000       23.6906  0.000000E+00       2.17389  0.000000E+00       2.17389  0.000000E+00  0.000000E+00      -4.34778       52.1585       1.51622        1       Scatag_D
      72       845.509       4310.45  0.000000E+00  0.000000E+00       2201.05  0.000000E+00       1971.95       2027.66  0.000000E+00       8273.16       40.0496       14531.3 -1.000000E+10 -1.000000E+10 -1.000000E+10 -1.000000E+10 -1.000000E+10  0.000000E+00  0.000000E+00  0.000000E+00  0.000000E+00  0.000000E+00  0.000000E+00  0.000000E+00  0.000000E+00  0.000000E+00  0.000000E+00  0.000000E+00  0.000000E+00       Funtag_D
      73  2.557449E+05  2.217310E+05       34013.8       21.0370      0.195570       1.15529      0.224568      0.242732    -0.0448126    -0.0610222    0.00546575      0.888748       Opttag_D
      73       52.9501       3.03387       1.00000  0.000000E+00       1.00000  0.000000E+00      0.830437       52.5367       1.00000       23.6722  0.000000E+00       2.16381  0.000000E+00       2.16381  0.000000E+00  0.000000E+00      -4.32762       52.1585       1.51622        1       Scatag_D
      73       838.686       4304.46  0.000000E+00  0.000000E+00       2199.06  0.000000E+00       1972.36       2024.53  0.000000E+00       8238.96       40.4909       14395.3 -1.000000E+10 -1.000000E+10 -1.000000E+10 -1.000000E+10 -1.000000E+10  0.000000E+00  0.000000E+00  0.000000E+00  0.000000E+00  0.000000E+00  0.000000E+00  0.000000E+00  0.000000E+00  0.000000E+00  0.000000E+00  0.000000E+00  0.000000E+00       Funtag_D
      74  2.556602E+05  2.217319E+05       33928.3       24.5983      0.195647       1.15382      0.224571      0.242732    -0.0448176    -0.0610360    0.00543301      0.888087       Opttag_D
      74       52.9539       3.03463       1.00000  0.000000E+00       1.00000  0.000000E+00      0.830434       52.5373       1.00000       23.6712  0.000000E+00       2.16398  0.000000E+00       2.16398  0.000000E+00  0.000000E+00      -4.32797       52.1585       1.51622        1       Scatag_D
      74       828.668       4298.06  0.000000E+00  0.000000E+00       2198.31  0.000000E+00       1962.40       2023.56  0.000000E+00       8234.59       40.6831       14342.0 -1.000000E+10 -1.000000E+10 -1.000000E+10 -1.000000E+10 -1.000000E+10  0.000000E+00  0.000000E+00  0.000000E+00  0.000000E+00  0.000000E+00  0.000000E+00  0.000000E+00  0.000000E+00  0.000000E+00  0.000000E+00  0.000000E+00  0.000000E+00       Funtag_D
      75  2.555902E+05  2.217285E+05       33861.7       13.4125      0.195315       1.14801      0.224569      0.242701    -0.0447985    -0.0609736    0.00540806      0.886387       Opttag_D
      75       52.9415       3.03200       1.00000  0.000000E+00       1.00000  0.000000E+00      0.830409       52.5295       1.00000       23.6726  0.000000E+00       2.16565  0.000000E+00       2.16565  0.000000E+00  0.000000E+00      -4.33130       52.1585       1.51622        1       Scatag_D
      75       821.076       4281.62  0.000000E+00  0.000000E+00       2198.08  0.000000E+00       1956.99       1997.07  0.000000E+00       8237.07       40.6552       14329.2 -1.000000E+10 -1.000000E+10 -1.000000E+10 -1.000000E+10 -1.000000E+10  0.000000E+00  0.000000E+00  0.000000E+00  0.000000E+00  0.000000E+00  0.000000E+00  0.000000E+00  0.000000E+00  0.000000E+00  0.000000E+00  0.000000E+00  0.000000E+00       Funtag_D
      76  2.555062E+05  2.217300E+05       33776.2       10.9180      0.195717       1.15075      0.224571      0.242721    -0.0448070    -0.0610263    0.00540129      0.885313       Opttag_D
      76       52.9580       3.03502       1.00000  0.000000E+00       1.00000  0.000000E+00      0.830419       52.5380       1.00000       23.6722  0.000000E+00       2.16401  0.000000E+00       2.16401  0.000000E+00  0.000000E+00      -4.32803       52.1585       1.51622        1       Scatag_D
      76       819.020       4271.25  0.000000E+00  0.000000E+00       2196.64  0.000000E+00       1947.90       1989.03  0.000000E+00       8229.72       40.5688       14282.1 -1.000000E+10 -1.000000E+10 -1.000000E+10 -1.000000E+10 -1.000000E+10  0.000000E+00  0.000000E+00  0.000000E+00  0.000000E+00  0.000000E+00  0.000000E+00  0.000000E+00  0.000000E+00  0.000000E+00  0.000000E+00  0.000000E+00  0.000000E+00       Funtag_D
      77  2.554399E+05  2.217330E+05       33706.9       13.6304      0.196315       1.17076      0.224571      0.242758    -0.0448237    -0.0611087    0.00540716      0.884633       Opttag_D
      77       52.9818       3.03946       1.00000  0.000000E+00       1.00000  0.000000E+00      0.830440       52.5503       1.00000       23.6719  0.000000E+00       2.16144  0.000000E+00       2.16144  0.000000E+00  0.000000E+00      -4.32287       52.1585       1.51622        1       Scatag_D
      77       820.803       4264.69  0.000000E+00  0.000000E+00       2194.87  0.000000E+00       1939.79       1991.23  0.000000E+00       8220.11       40.4546       14235.0 -1.000000E+10 -1.000000E+10 -1.000000E+10 -1.000000E+10 -1.000000E+10  0.000000E+00  0.000000E+00  0.000000E+00  0.000000E+00  0.000000E+00  0.000000E+00  0.000000E+00  0.000000E+00  0.000000E+00  0.000000E+00  0.000000E+00  0.000000E+00       Funtag_D
      78  2.553678E+05  2.217406E+05       33627.2       24.6675      0.197768       1.25102      0.224572      0.242839    -0.0448660    -0.0612810    0.00542902      0.884203       Opttag_D
      78       53.0379       3.04992       1.00000  0.000000E+00       1.00000  0.000000E+00      0.830495       52.5759       1.00000       23.6757  0.000000E+00       2.15548  0.000000E+00       2.15548  0.000000E+00  0.000000E+00      -4.31095       52.1585       1.51622        1       Scatag_D
      78       827.453       4260.55  0.000000E+00  0.000000E+00       2191.54  0.000000E+00       1932.05       2011.12  0.000000E+00       8198.03       40.3403       14166.1 -1.000000E+10 -1.000000E+10 -1.000000E+10 -1.000000E+10 -1.000000E+10  0.000000E+00  0.000000E+00  0.000000E+00  0.000000E+00  0.000000E+00  0.000000E+00  0.000000E+00  0.000000E+00  0.000000E+00  0.000000E+00  0.000000E+00  0.000000E+00       Funtag_D
      79  2.552825E+05  2.217466E+05       33535.9       12.8870      0.198402       1.28605      0.224577      0.242883    -0.0448992    -0.0613868    0.00541553      0.882638       Opttag_D
      79       53.0622       3.05464       1.00000  0.000000E+00       1.00000  0.000000E+00      0.830522       52.5870       1.00000       23.6771  0.000000E+00       2.15294  0.000000E+00       2.15294  0.000000E+00  0.000000E+00      -4.30589       52.1585       1.51622        1       Scatag_D
      79       823.346       4245.47  0.000000E+00  0.000000E+00       2190.17  0.000000E+00       1932.22       1980.44  0.000000E+00       8187.15       40.2381       14136.9 -1.000000E+10 -1.000000E+10 -1.000000E+10 -1.000000E+10 -1.000000E+10  0.000000E+00  0.000000E+00  0.000000E+00  0.000000E+00  0.000000E+00  0.000000E+00  0.000000E+00  0.000000E+00  0.000000E+00  0.000000E+00  0.000000E+00  0.000000E+00       Funtag_D
      80  2.552239E+05  2.217459E+05       33478.1       9.64638      0.198414       1.29005      0.224576      0.242876    -0.0448953    -0.0613862    0.00538560      0.881722       Opttag_D
      80       53.0638       3.05468       1.00000  0.000000E+00       1.00000  0.000000E+00      0.830521       52.5843       1.00000       23.6791  0.000000E+00       2.15291  0.000000E+00       2.15291  0.000000E+00  0.000000E+00      -4.30582       52.1585       1.51622        1       Scatag_D
      80       814.270       4236.66  0.000000E+00  0.000000E+00       2189.88  0.000000E+00       1935.35       1973.68  0.000000E+00       8183.28       40.2379       14104.7 -1.000000E+10 -1.000000E+10 -1.000000E+10 -1.000000E+10 -1.000000E+10  0.000000E+00  0.000000E+00  0.000000E+00  0.000000E+00  0.000000E+00  0.000000E+00  0.000000E+00  0.000000E+00  0.000000E+00  0.000000E+00  0.000000E+00  0.000000E+00       Funtag_D
      81  2.551714E+05  2.217493E+05       33422.1       9.81207      0.198781       1.31301      0.224579      0.242898    -0.0449143    -0.0614406    0.00536797      0.881072       Opttag_D
      81       53.0794       3.05752       1.00000  0.000000E+00       1.00000  0.000000E+00      0.830535       52.5873       1.00000       23.6822  0.000000E+00       2.15170  0.000000E+00       2.15170  0.000000E+00  0.000000E+00      -4.30341       52.1585       1.51622        1       Scatag_D
      81       808.948       4230.43  0.000000E+00  0.000000E+00       2188.77  0.000000E+00       1937.62       1971.61  0.000000E+00       8175.12       40.1955       14069.4 -1.000000E+10 -1.000000E+10 -1.000000E+10 -1.000000E+10 -1.000000E+10  0.000000E+00  0.000000E+00  0.000000E+00  0.000000E+00  0.000000E+00  0.000000E+00  0.000000E+00  0.000000E+00  0.000000E+00  0.000000E+00  0.000000E+00  0.000000E+00       Funtag_D
      82  2.551347E+05  2.217604E+05       33374.3       26.3554      0.199818       1.36651      0.224590      0.242975    -0.0449763    -0.0616098    0.00536163      0.880683       Opttag_D
      82       53.1217       3.06547       1.00000  0.000000E+00       1.00000  0.000000E+00      0.830577       52.5963       1.00000       23.6884  0.000000E+00       2.14857  0.000000E+00       2.14857  0.000000E+00  0.000000E+00      -4.29714       52.1585       1.51622        1       Scatag_D
      82       807.038       4226.69  0.000000E+00  0.000000E+00       2186.03  0.000000E+00       1935.63       2000.52  0.000000E+00       8158.52       40.0552       14019.8 -1.000000E+10 -1.000000E+10 -1.000000E+10 -1.000000E+10 -1.000000E+10  0.000000E+00  0.000000E+00  0.000000E+00  0.000000E+00  0.000000E+00  0.000000E+00  0.000000E+00  0.000000E+00  0.000000E+00  0.000000E+00  0.000000E+00  0.000000E+00       Funtag_D
      83  2.550634E+05  2.217629E+05       33300.6       10.6122      0.199969       1.37135      0.224592      0.242993    -0.0449899    -0.0616463    0.00534785      0.880003       Opttag_D
      83       53.1289       3.06690       1.00000  0.000000E+00       1.00000  0.000000E+00      0.830583       52.5984       1.00000       23.6881  0.000000E+00       2.14823  0.000000E+00       2.14823  0.000000E+00  0.000000E+00      -4.29646       52.1585       1.51622        1       Scatag_D
      83       802.896       4220.16  0.000000E+00  0.000000E+00       2185.49  0.000000E+00       1933.72       1958.62  0.000000E+00       8155.66       40.0498       14004.0 -1.000000E+10 -1.000000E+10 -1.000000E+10 -1.000000E+10 -1.000000E+10  0.000000E+00  0.000000E+00  0.000000E+00  0.000000E+00  0.000000E+00  0.000000E+00  0.000000E+00  0.000000E+00  0.000000E+00  0.000000E+00  0.000000E+00  0.000000E+00       Funtag_D
      84  2.550187E+05  2.217677E+05       33250.9       8.58942      0.200352       1.38328      0.224596      0.243034    -0.0450170    -0.0617267    0.00534316      0.879564       Opttag_D
      84       53.1450       3.07009       1.00000  0.000000E+00       1.00000  0.000000E+00      0.830601       52.6041       1.00000       23.6874  0.000000E+00       2.14718  0.000000E+00       2.14718  0.000000E+00  0.000000E+00      -4.29437       52.1585       1.51622        1       Scatag_D
      84       801.488       4215.96  0.000000E+00  0.000000E+00       2184.47  0.000000E+00       1928.19       1950.97  0.000000E+00       8149.99       40.0509       13979.8 -1.000000E+10 -1.000000E+10 -1.000000E+10 -1.000000E+10 -1.000000E+10  0.000000E+00  0.000000E+00  0.000000E+00  0.000000E+00  0.000000E+00  0.000000E+00  0.000000E+00  0.000000E+00  0.000000E+00  0.000000E+00  0.000000E+00  0.000000E+00       Funtag_D
      85  2.549730E+05  2.217744E+05       33198.6       10.7841      0.200985       1.40141      0.224598      0.243097    -0.0450544    -0.0618564    0.00534835      0.879059       Opttag_D
      85       53.1710       3.07512       1.00000  0.000000E+00       1.00000  0.000000E+00      0.830631       52.6121       1.00000       23.6866  0.000000E+00       2.14538  0.000000E+00       2.14538  0.000000E+00  0.000000E+00      -4.29076       52.1585       1.51622        1       Scatag_D
      85       803.046       4211.12  0.000000E+00  0.000000E+00       2182.86  0.000000E+00       1921.58       1950.72  0.000000E+00       8141.59       40.1175       13947.6 -1.000000E+10 -1.000000E+10 -1.000000E+10 -1.000000E+10 -1.000000E+10  0.000000E+00  0.000000E+00  0.000000E+00  0.000000E+00  0.000000E+00  0.000000E+00  0.000000E+00  0.000000E+00  0.000000E+00  0.000000E+00  0.000000E+00  0.000000E+00       Funtag_D
      86  2.548916E+05  2.217827E+05       33108.9       19.0006      0.202191       1.42114      0.224592      0.243213    -0.0451005    -0.0620986    0.00535543      0.878219       Opttag_D
      86       53.2206       3.08438       1.00000  0.000000E+00       1.00000  0.000000E+00      0.830692       52.6281       1.00000       23.6824  0.000000E+00       2.14162  0.000000E+00       2.14162  0.000000E+00  0.000000E+00      -4.28325       52.1585       1.51622        1       Scatag_D
      86       805.173       4203.07  0.000000E+00  0.000000E+00       2179.83  0.000000E+00       1914.24       1958.47  0.000000E+00       8127.24       40.2771       13880.6 -1.000000E+10 -1.000000E+10 -1.000000E+10 -1.000000E+10 -1.000000E+10  0.000000E+00  0.000000E+00  0.000000E+00  0.000000E+00  0.000000E+00  0.000000E+00  0.000000E+00  0.000000E+00  0.000000E+00  0.000000E+00  0.000000E+00  0.000000E+00       Funtag_D
      87  2.548251E+05  2.218000E+05       33025.1       18.2888      0.204014       1.44569      0.224594      0.243391    -0.0451968    -0.0625047    0.00537619      0.877016       Opttag_D
      87       53.2919       3.09824       1.00000  0.000000E+00       1.00000  0.000000E+00      0.830789       52.6508       1.00000       23.6776  0.000000E+00       2.13622  0.000000E+00       2.13622  0.000000E+00  0.000000E+00      -4.27243       52.1585       1.51622        1       Scatag_D
      87       811.428       4191.57  0.000000E+00  0.000000E+00       2175.99  0.000000E+00       1909.93       1958.00  0.000000E+00       8112.64       40.5391       13825.0 -1.000000E+10 -1.000000E+10 -1.000000E+10 -1.000000E+10 -1.000000E+10  0.000000E+00  0.000000E+00  0.000000E+00  0.000000E+00  0.000000E+00  0.000000E+00  0.000000E+00  0.000000E+00  0.000000E+00  0.000000E+00  0.000000E+00  0.000000E+00       Funtag_D
      88  2.547569E+05  2.217925E+05       32964.4       9.39441      0.203886       1.43113      0.224581      0.243385    -0.0451552    -0.0624753    0.00534777      0.876225       Opttag_D
      88       53.2884       3.09696       1.00000  0.000000E+00       1.00000  0.000000E+00      0.830785       52.6495       1.00000       23.6753  0.000000E+00       2.13597  0.000000E+00       2.13597  0.000000E+00  0.000000E+00      -4.27194       52.1585       1.51622        1       Scatag_D
      88       802.870       4184.00  0.000000E+00  0.000000E+00       2175.89  0.000000E+00       1912.79       1939.51  0.000000E+00       8112.47       40.2576       13796.6 -1.000000E+10 -1.000000E+10 -1.000000E+10 -1.000000E+10 -1.000000E+10  0.000000E+00  0.000000E+00  0.000000E+00  0.000000E+00  0.000000E+00  0.000000E+00  0.000000E+00  0.000000E+00  0.000000E+00  0.000000E+00  0.000000E+00  0.000000E+00       Funtag_D
      89  2.546938E+05  2.217840E+05       32909.8       10.6558      0.203955       1.40857      0.224561      0.243407    -0.0451078    -0.0624997    0.00531859      0.875308       Opttag_D
      89       53.2934       3.09714       1.00000  0.000000E+00       1.00000  0.000000E+00      0.830794       52.6529       1.00000       23.6701  0.000000E+00       2.13480  0.000000E+00       2.13480  0.000000E+00  0.000000E+00      -4.26959       52.1585       1.51622        1       Scatag_D
      89       794.134       4175.26  0.000000E+00  0.000000E+00       2175.19  0.000000E+00       1918.06       1948.06  0.000000E+00       8110.95       39.8693       13748.3 -1.000000E+10 -1.000000E+10 -1.000000E+10 -1.000000E+10 -1.000000E+10  0.000000E+00  0.000000E+00  0.000000E+00  0.000000E+00  0.000000E+00  0.000000E+00  0.000000E+00  0.000000E+00  0.000000E+00  0.000000E+00  0.000000E+00  0.000000E+00       Funtag_D
      90  2.546396E+05  2.217829E+05       32856.7       13.5010      0.204523       1.39200      0.224546      0.243480    -0.0451015    -0.0626432    0.00530348      0.874755       Opttag_D
      90       53.3169       3.10127       1.00000  0.000000E+00       1.00000  0.000000E+00      0.830832       52.6640       1.00000       23.6640  0.000000E+00       2.13227  0.000000E+00       2.13227  0.000000E+00  0.000000E+00      -4.26453       52.1585       1.51622        1       Scatag_D
      90       789.628       4169.98  0.000000E+00  0.000000E+00       2173.66  0.000000E+00       1921.21       1959.06  0.000000E+00       8106.60       39.5125       13697.1 -1.000000E+10 -1.000000E+10 -1.000000E+10 -1.000000E+10 -1.000000E+10  0.000000E+00  0.000000E+00  0.000000E+00  0.000000E+00  0.000000E+00  0.000000E+00  0.000000E+00  0.000000E+00  0.000000E+00  0.000000E+00  0.000000E+00  0.000000E+00       Funtag_D
      91  2.545669E+05  2.217919E+05       32775.1       12.3865      0.205867       1.37436      0.224538      0.243636    -0.0451515    -0.0629828    0.00529250      0.874496       Opttag_D
      91       53.3683       3.11121       1.00000  0.000000E+00       1.00000  0.000000E+00      0.830916       52.6860       1.00000       23.6573  0.000000E+00       2.12805  0.000000E+00       2.12805  0.000000E+00  0.000000E+00      -4.25610       52.1585       1.51622        1       Scatag_D
      91       786.361       4167.51  0.000000E+00  0.000000E+00       2170.89  0.000000E+00       1921.87       1956.51  0.000000E+00       8098.19       39.0697       13634.7 -1.000000E+10 -1.000000E+10 -1.000000E+10 -1.000000E+10 -1.000000E+10  0.000000E+00  0.000000E+00  0.000000E+00  0.000000E+00  0.000000E+00  0.000000E+00  0.000000E+00  0.000000E+00  0.000000E+00  0.000000E+00  0.000000E+00  0.000000E+00       Funtag_D
      92  2.545096E+05  2.218005E+05       32709.1       10.4253      0.206628       1.37036      0.224541      0.243721    -0.0451997    -0.0631778    0.00528166      0.874297       Opttag_D
      92       53.3955       3.11677       1.00000  0.000000E+00       1.00000  0.000000E+00      0.830962       52.6948       1.00000       23.6569  0.000000E+00       2.12632  0.000000E+00       2.12632  0.000000E+00  0.000000E+00      -4.25264       52.1585       1.51622        1       Scatag_D
      92       783.144       4165.62  0.000000E+00  0.000000E+00       2169.56  0.000000E+00       1915.74       1947.02  0.000000E+00       8091.74       39.0967       13597.2 -1.000000E+10 -1.000000E+10 -1.000000E+10 -1.000000E+10 -1.000000E+10  0.000000E+00  0.000000E+00  0.000000E+00  0.000000E+00  0.000000E+00  0.000000E+00  0.000000E+00  0.000000E+00  0.000000E+00  0.000000E+00  0.000000E+00  0.000000E+00       Funtag_D
      93  2.544599E+05  2.218136E+05       32646.3       10.3964      0.207535       1.37138      0.224553      0.243820    -0.0452725    -0.0634061    0.00528476      0.874157       Opttag_D
      93       53.4266       3.12338       1.00000  0.000000E+00       1.00000  0.000000E+00      0.831015       52.7035       1.00000       23.6593  0.000000E+00       2.12490  0.000000E+00       2.12490  0.000000E+00  0.000000E+00      -4.24980       52.1585       1.51622        1       Scatag_D
      93       784.064       4164.28  0.000000E+00  0.000000E+00       2168.16  0.000000E+00       1905.03       1947.16  0.000000E+00       8083.34       39.4717       13554.8 -1.000000E+10 -1.000000E+10 -1.000000E+10 -1.000000E+10 -1.000000E+10  0.000000E+00  0.000000E+00  0.000000E+00  0.000000E+00  0.000000E+00  0.000000E+00  0.000000E+00  0.000000E+00  0.000000E+00  0.000000E+00  0.000000E+00  0.000000E+00       Funtag_D
      94  2.544221E+05  2.218169E+05       32605.2       9.17098      0.207974       1.37055      0.224555      0.243861    -0.0452908    -0.0635012    0.00528501      0.873936       Opttag_D
      94       53.4411       3.12608       1.00000  0.000000E+00       1.00000  0.000000E+00      0.831038       52.7052       1.00000       23.6626  0.000000E+00       2.12424  0.000000E+00       2.12424  0.000000E+00  0.000000E+00      -4.24849       52.1585       1.51622        1       Scatag_D
      94       784.137       4162.17  0.000000E+00  0.000000E+00       2167.47  0.000000E+00       1899.71       1946.06  0.000000E+00       8077.87       39.7318       13528.0 -1.000000E+10 -1.000000E+10 -1.000000E+10 -1.000000E+10 -1.000000E+10  0.000000E+00  0.000000E+00  0.000000E+00  0.000000E+00  0.000000E+00  0.000000E+00  0.000000E+00  0.000000E+00  0.000000E+00  0.000000E+00  0.000000E+00  0.000000E+00       Funtag_D
      95  2.543682E+05  2.218196E+05       32548.6       9.69852      0.208835       1.36570      0.224552      0.243946    -0.0453061    -0.0636749    0.00529023      0.873801       Opttag_D
      95       53.4691       3.13091       1.00000  0.000000E+00       1.00000  0.000000E+00      0.831081       52.7072       1.00000       23.6700  0.000000E+00       2.12239  0.000000E+00       2.12239  0.000000E+00  0.000000E+00      -4.24478       52.1585       1.51622        1       Scatag_D
      95       785.687       4160.89  0.000000E+00  0.000000E+00       2166.06  0.000000E+00       1894.47       1949.08  0.000000E+00       8068.77       39.9679       13483.7 -1.000000E+10 -1.000000E+10 -1.000000E+10 -1.000000E+10 -1.000000E+10  0.000000E+00  0.000000E+00  0.000000E+00  0.000000E+00  0.000000E+00  0.000000E+00  0.000000E+00  0.000000E+00  0.000000E+00  0.000000E+00  0.000000E+00  0.000000E+00       Funtag_D
      96  2.543343E+05  2.218242E+05       32510.0       19.2328      0.210368       1.35557      0.224546      0.244094    -0.0453317    -0.0639491    0.00530466      0.873916       Opttag_D
      96       53.5182       3.13954       1.00000  0.000000E+00       1.00000  0.000000E+00      0.831152       52.7196       1.00000       23.6828  0.000000E+00       2.11811  0.000000E+00       2.11811  0.000000E+00  0.000000E+00      -4.23621       52.1585       1.51622        1       Scatag_D
      96       789.979       4161.99  0.000000E+00  0.000000E+00       2163.62  0.000000E+00       1893.57       1979.17  0.000000E+00       8057.74       39.8142       13424.2 -1.000000E+10 -1.000000E+10 -1.000000E+10 -1.000000E+10 -1.000000E+10  0.000000E+00  0.000000E+00  0.000000E+00  0.000000E+00  0.000000E+00  0.000000E+00  0.000000E+00  0.000000E+00  0.000000E+00  0.000000E+00  0.000000E+00  0.000000E+00       Funtag_D
      97  2.542848E+05  2.218226E+05       32462.2       8.89022      0.210662       1.35381      0.224543      0.244129    -0.0453226    -0.0640015    0.00529001      0.873368       Opttag_D
      97       53.5273       3.14081       1.00000  0.000000E+00       1.00000  0.000000E+00      0.831161       52.7204       1.00000       23.6875  0.000000E+00       2.11678  0.000000E+00       2.11678  0.000000E+00  0.000000E+00      -4.23357       52.1585       1.51622        1       Scatag_D
      97       785.622       4156.77  0.000000E+00  0.000000E+00       2163.04  0.000000E+00       1894.76       1958.94  0.000000E+00       8056.58       39.5249       13407.0 -1.000000E+10 -1.000000E+10 -1.000000E+10 -1.000000E+10 -1.000000E+10  0.000000E+00  0.000000E+00  0.000000E+00  0.000000E+00  0.000000E+00  0.000000E+00  0.000000E+00  0.000000E+00  0.000000E+00  0.000000E+00  0.000000E+00  0.000000E+00       Funtag_D
      98  2.542487E+05  2.218210E+05       32427.7       8.79631      0.211062       1.35227      0.224536      0.244175    -0.0453140    -0.0640679    0.00527072      0.872801       Opttag_D
      98       53.5398       3.14265       1.00000  0.000000E+00       1.00000  0.000000E+00      0.831172       52.7234       1.00000       23.6938  0.000000E+00       2.11486  0.000000E+00       2.11486  0.000000E+00  0.000000E+00      -4.22973       52.1585       1.51622        1       Scatag_D
      98       779.901       4151.37  0.000000E+00  0.000000E+00       2162.22  0.000000E+00       1896.81       1960.73  0.000000E+00       8055.50       39.0816       13382.1 -1.000000E+10 -1.000000E+10 -1.000000E+10 -1.000000E+10 -1.000000E+10  0.000000E+00  0.000000E+00  0.000000E+00  0.000000E+00  0.000000E+00  0.000000E+00  0.000000E+00  0.000000E+00  0.000000E+00  0.000000E+00  0.000000E+00  0.000000E+00       Funtag_D
      99  2.542082E+05  2.218232E+05       32384.9       10.3061      0.211898       1.35133      0.224540      0.244267    -0.0453262    -0.0642292    0.00526350      0.872187       Opttag_D
      99       53.5650       3.14690       1.00000  0.000000E+00       1.00000  0.000000E+00      0.831199       52.7293       1.00000       23.7063  0.000000E+00       2.11195  0.000000E+00       2.11195  0.000000E+00  0.000000E+00      -4.22389       52.1585       1.51622        1       Scatag_D
      99       777.766       4145.53  0.000000E+00  0.000000E+00       2160.67  0.000000E+00       1898.29       1968.86  0.000000E+00       8052.12       38.7196       13343.0 -1.000000E+10 -1.000000E+10 -1.000000E+10 -1.000000E+10 -1.000000E+10  0.000000E+00  0.000000E+00  0.000000E+00  0.000000E+00  0.000000E+00  0.000000E+00  0.000000E+00  0.000000E+00  0.000000E+00  0.000000E+00  0.000000E+00  0.000000E+00       Funtag_D

   MaxLik Scaling Cycle 0105

   Wilson plot

   Statistics in resolution bins after refinement cycle 0003
```


|  |  |
| --- | --- |
|  | - Rxpct factors plot- Rxpct factors table- <Log-Lik Gain>- Ln Sigma A- Correlation Coefficients |


```
   Iters     Tot_Funct    Xray_Funct    Geom_Funct          Grms     rmsD2init     maxD2init             R         Rfree           LLG       LLGfree     rms(bond)    rms(angle)
   Iters             K   B_IMPF_FRAG        K_MISS   B_TEMP_MISS   K_IMPF_MISS   B_IMPF_MISS        K_SOLV   B_TEMP_SOLV   K_IMPF_SOLV   B_IMPF_SOLV        B_TEMP     B_TEMP_11     B_TEMP_12     B_TEMP_22     B_TEMP_13     B_TEMP_23     B_TEMP_33      K_WILSON      B_WILSON      FGCYCLES
   Iters          BOND         ANGLE      IMPROPER       TORSION    SINTORSION      TRIGONAL        PLANAR  BCORRELATION       CONTACT     IDEALDIST        CHIRAL    SIMILARITY       OCCSSUM  UTILDISTANCE     UTILANGLE   UTILTORSION        RHOFIT        ADP-01        ADP-02        ADP-03        ADP-04        ADP-05        ADP-06        ADP-07        ADP-08        ADP-09        ADP-10      RHOFITPC            QM
     100  2.541638E+05  2.218314E+05       32332.4       23.9260      0.213399       1.36245      0.224555      0.244446    -0.0453717    -0.0645636    0.00526444      0.870868       Opttag_D
     100       53.6072       3.15417       1.00000  0.000000E+00       1.00000  0.000000E+00      0.831235       52.7314       1.00000       23.7353  0.000000E+00       2.10784  0.000000E+00       2.10784  0.000000E+00  0.000000E+00      -4.21568       52.1585       1.51622        1       Scatag_D
     100       778.046       4133.00  0.000000E+00  0.000000E+00       2157.73  0.000000E+00       1896.97       2012.59  0.000000E+00       8044.03       38.7024       13271.3 -1.000000E+10 -1.000000E+10 -1.000000E+10 -1.000000E+10 -1.000000E+10  0.000000E+00  0.000000E+00  0.000000E+00  0.000000E+00  0.000000E+00  0.000000E+00  0.000000E+00  0.000000E+00  0.000000E+00  0.000000E+00  0.000000E+00  0.000000E+00       Funtag_D
   Geometry Restraint Deviations SCREEN Information

 
 No -bcorrel_zeroocchydrogens_factor flag found - keeping default: will set B for OCC=0 hydrogens to    1.200 times heavy-atom B


 TLS information. Total number of groups:     2


 Group   Natoms    FixRB   FixTLS   Group Tag    Group Spec
     1     2770      Y        Y     tls1    { A|* }
     2     2767      Y        Y     tls2    { B|* }
 
NOTE BUSTER_TLS_SET tls1 { A|* }
NOTE BUSTER_TLS_T   tls1     -1.39020E-01 -5.95499E-02  2.01158E-01 -1.38376E-02  3.83768E-02  4.73733E-03
NOTE BUSTER_TLS_L   tls1      6.24652E-01  3.28075E-01  9.50159E-02  2.42930E-01 -1.55227E-01  5.25158E-02
NOTE BUSTER_TLS_S   tls1      1.76373E-01 -1.14237E-01  1.11924E-01 -5.94811E-02 -8.26876E-02 -2.72786E-02 -4.25898E-02  4.14621E-03
NOTE BUSTER_TLS_O   tls1     -2.33295E+01 -8.34950E+01  1.14606E+01
NOTE BUSTER_TLS_FIX tls1 ALL
 
NOTE BUSTER_TLS_SET tls2 { B|* }
NOTE BUSTER_TLS_T   tls2     -1.42976E-01 -9.83390E-02  2.69975E-01  9.37908E-03  3.33279E-02  5.70942E-03
NOTE BUSTER_TLS_L   tls2      5.22272E-01  2.80406E-01  2.72933E-01  2.71705E-01 -2.54092E-01 -2.50673E-01
NOTE BUSTER_TLS_S   tls2      1.43174E-03  8.29114E-02 -4.40524E-02  5.30728E-02  1.26218E-01 -2.00028E-02 -3.40003E-02  3.11097E-02
NOTE BUSTER_TLS_O   tls2      2.19831E+01 -7.17259E+01  3.45078E+00
NOTE BUSTER_TLS_FIX tls2 ALL

 For final position, Geometry function summary
     term                           number     weight       rms        GooF      function     fn/numb
     ================================================================================================
     BOND bond lengths (angs)       5652       2.000      0.0053       0.262       778.05       0.138
     ANGL bond angles (degs)        7670       2.000        0.87       0.519      4133.00       0.539
     TORS split-harmonic tors       1265       0.000       16.80       1.058         0.00       0.000
     SINTOR sinusoidal tors         1265       2.000       16.80                  2157.73       1.706
     PLAN general plane (angs)       963       5.000      0.0126       0.628      1896.97       1.970
     BCORrelations (angs^2)         5652      10.000       0.529       0.189      2012.59       0.356
     CONT (bad contacts)               0       5.000                                 0.00
     IDEAL(ideal-dist contact)      5066       4.000                              8044.03
     CHIRAL (gelly semiharmon)       740       5.000                                38.70
     SIM similarity restraint for NCS and/or TARGET                              13271.28
     OCCSUM restraint on sum of occupanies                                           0.00       as none defined
     DISTAN utility restraints interatomic distance                                  0.00       as none defined
     UTILANGLE utility angle restraints                                              0.00       as none defined
     UTILTOR utility restraints on torsion angles                                    0.00       as none defined
     Peptide omega torsion angles                           2.67 degs - monitor only
 
 WARNING: Have |delta/sigma| deviations > 5.0 sigma. Number of outliers for each term: 
 WARNING:         4 bond angles.  Worst is     5.1 sigs   134.40 degs A|28:C.B(ASN)=A|29:N(ASN)=A|29:CA(ASN)

 Weighted rms      0.0053         0.871  (temporary rms bond and angle for BUSTER to pick up!)
 Weighted intra-residual rms(bond) for StdProtein (excluding H)      0.0051
 Weighted intra-residual rms(bond) for Buffer     (excluding H)      0.0036
 Outputing geometry progress graph in plotmtv format to file:Geom_Res.mtv

 ############### Running final geometry/screen analysis (screen_final.txt)

 s/r gelly normal termination
```

Statistics and scale factors vs. cycle number
```

|  |  |  |
| --- | --- | --- |
|  | - Rxpct factors- <Log-Lik Gain>- GEOM Residuals- Ln Sigma_A_ | - Scale Factors- B Factors- Imperfection Factors |

```
   Final Geometry Information


---


### 2. Final results

Statistics and scale factors vs. cycle number
```

|  |  |  |
| --- | --- | --- |
|  | - Rxpct factors- <Log-Lik Gain>- GEOM Residuals- Ln Sigma_A_ | - Scale Factors- B Factors- Imperfection Factors |

```
   Average Figure of Merit
   Structure Factor Amplitudes


 # normal termination

  Program stopped.
```

closesok message: time spent in tree = 1732.80 (user) 40.51 (sys)

---

# Normal termination

```
 Resources usage summary:
     Time spent in client ...................  1732.81 (user)       40.52 (system)
     Time spent in scripts (    27 calls) ...     3.60 (user)        0.19 (system)
     Time spent in server ...................     0.00 (user)        0.00 (system)

     Max resident memory used (kb) .......... 519100
     Number of page faults .................. 0 (major)    920120 (minor)
     Number of context switches ............. 1118805 (niced)    7658 (other)
```
